# Green steel from red mud through climate-neutral hydrogen plasma reduction

**DOI:** 10.1038/s41586-023-06901-z

**Published:** 2024-01-24

**Authors:** Matic Jovičević-Klug, Isnaldi R. Souza Filho, Hauke Springer, Christian Adam, Dierk Raabe

**Affiliations:** 1 Max-Planck-Institut für Eisenforschung, Düsseldorf, Germany; 2 Institut für Bildsame Formgebung, RWTH Aachen University, Aachen, Germany; 3 Bundesanstalt für Materialforschung und -prüfung, Berlin, Germany

**Keywords:** Materials science, Metals and alloys

## Abstract

Red mud is the waste of bauxite refinement into alumina, the feedstock for aluminium production^1^. With about 180 million tonnes produced per year^1^, red mud has amassed to one of the largest environmentally hazardous waste products, with the staggering amount of 4 billion tonnes accumulated on a global scale^1^. Here we present how this red mud can be turned into valuable and sustainable feedstock for ironmaking using fossil-free hydrogen-plasma-based reduction, thus mitigating a part of the steel-related carbon dioxide emissions by making it available for the production of several hundred million tonnes of green steel. The process proceeds through rapid liquid-state reduction, chemical partitioning, as well as density-driven and viscosity-driven separation between metal and oxides. We show the underlying chemical reactions, pH-neutralization processes and phase transformations during this surprisingly simple and fast reduction method. The approach establishes a sustainable toxic-waste treatment from aluminium production through using red mud as feedstock to mitigate greenhouse gas emissions from steelmaking.

## Abstract (web summary)

Red mud is shown to yield green steel through fossil-free hydrogen-plasma-based reduction, a simple and fast method involving rapid liquid-state reduction, chemical partitioning, and density-driven and viscosity-driven separation.

## Main

**Fig. 1:**
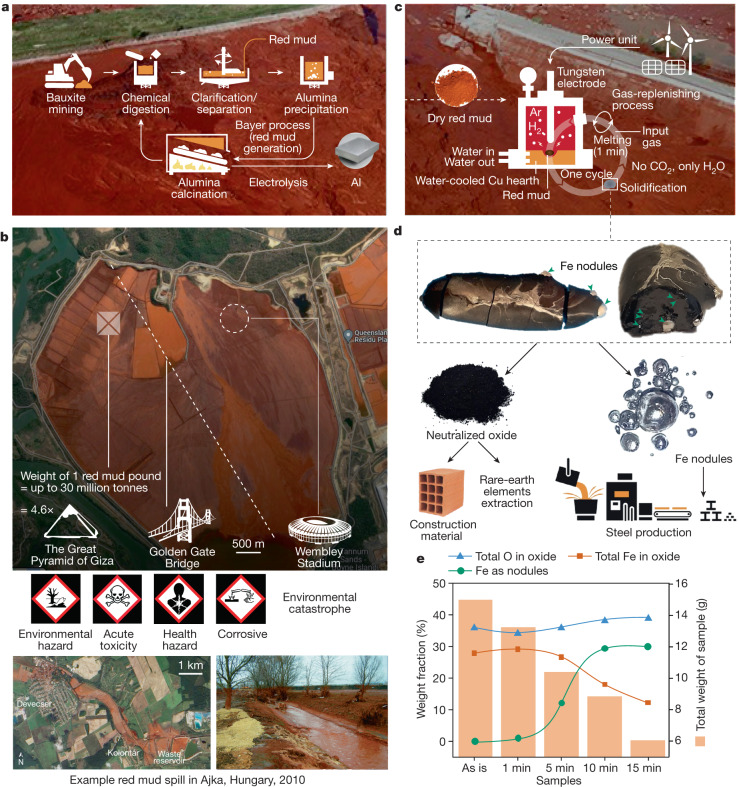
The generation, storage and hazards of red muds and solution with hydrogen plasma treatment. **a**, Schematic representation of the bauxite mining and subsequent Bayer process, in which the ore is chemically transformed into alumina, the feedstock material for aluminium production by means of electrolysis, and the waste red mud. **b**, Top, satellite image of a waste pond used to store red mud. Schematics of classical monuments are overlaid as a reference for the huge dimensions of such a reservoir. Bottom, satellite image (left) and photograph (right) showing catastrophic events when red mud dams break. **c**, Schematic representation of the hydrogen-plasma-based process used here to convert 15 g red mud portion into metallic iron. **d**, Example of red mud powder transformed into the final product after 10 min of reduction (solidified sample). The resulting sample was separated into remaining oxide-rich powder and iron nodules by mechanical crushing and magnetic separation. The oxide portions can be destined to civil construction ends and the iron to steel production. **e**, Diagram depicting the extracted weight change with reduction time in relation to the obtained Fe in the form of nodules, as well as the Fe and O content within the oxide portion of the samples. Credits: **b** (top), copyright TerraMetrics, LLC, www.terrametrics.com, imagery © 2022 Google, TerraMetrics, imagery © 2022 CNES/Airbus, Maxar Technologies, map data © 2022; **b** (bottom left, satellite image), © NASA Earth Observatory image created by Jesse Allen, using EO-1 ALI data provided courtesy of the NASA EO-1 team (source https://earthobservatory.nasa.gov/images/46360/toxic-sludge-in-hungary); **b**, (bottom right), reprinted (adapted) with permission from ref. ^28^, copyright (2023) American Chemical Society.

Aluminium is the fastest-growing mass-produced material group^1^, a prerequisite for lightweight design of vehicles, food containers and storage, as well as civil-engineering structures. Its synthesis is based on the refinement of a mixed mineral ore, called bauxite, into alumina by means of the Bayer process (Fig. 1a), leaving the colloquially named red mud as a by-product^1^. Also called red sludge or bauxite residue, this highly alkaline (pH 10–13) slurry contains a complex mixture of various oxides, traces of valuable metals (for example, Sc and Y), as well as potentially toxic heavy metals (Cd, Cr, V)^2,3^. Thus, the huge market growth for aluminium alloys translates into a rapidly growing surge of red mud, accumulating into a gigantic global stock of 4 billion tonnes^4^ at present. As an unwanted by-product, only 3% of its yearly produced quantity is recycled, mostly for construction, using costly neutralization processing^5^. Most of the red mud is usually simply disposed of in extremely large (see Fig. 1b) waste ponds, dried mounts or landfills, if not just poured into open nature^5,6^. This practice is not only very costly, representing 5% of the total aluminium production value^5^, but also causes severe and long-lasting environmental and humanitarian catastrophes^7^ by often irresponsible storage (Fig. 1b).

Red muds are geographically highly diverse in both quantity and chemical composition, which also influence their usability and capability to extract Fe through carbothermic smelting (more details in Supplementary information section ‘Geographic dependency of red mud and current state on utilising red mud for Fe extraction’). Several attempts have been made to replace C-carrying substances by hydrogen in the form of molecules (H_2_) or plasma as the reducing agent to extract iron from red mud while simultaneously avoiding CO_2_ production^8,9^. One of them suggests the use of hydrogen-plasma-smelting treatment to refine bauxite into alumina with a possibility of completely suppressing the production of red mud^10^. However, all the possibilities reported in refs. ^8–10^ inevitably require further preprocessing of the red mud through roasting, milling, pelletizing and wet magnetic separation. Such complex processing makes the red mud valorization financially unattractive and also does not help eliminate the direct emissions of CO_2_, as the intermediate stages require roasting and pelletizing, processes that are associated with high CO_2_ footprints. Therefore, simple, innovative and carbon-neutral single-step strategies are required to sustainably process and neutralize red mud, whilst extracting its valuable content for downstream manufacturing.

Replacing the carbon-based reducing substances by highly energetic hydrogen species contained in a hydrogen plasma is an energy-efficient and partially electrified pathway to extract green iron from its molten oxides in one single-step process^11^ (particularly if green hydrogen and renewable electricity are used^12^). Thus, huge opportunities exist to further exploit this emergent route to transform red mud into a sustainable feedstock for clean iron production. Here we show that the direct exposure of red mud to a lean hydrogen thermal plasma (Ar-10%H_2_) ignited in an electric arc furnace (EAF) allows for producing liquid iron without the need for any preceding treatment of the input material (Fig. 1c), different from previous studies^3,8–10,13,14^, which require energy-intensive pre-treatment and post-treatment of the materials.

**Extended Data Fig. 1:**
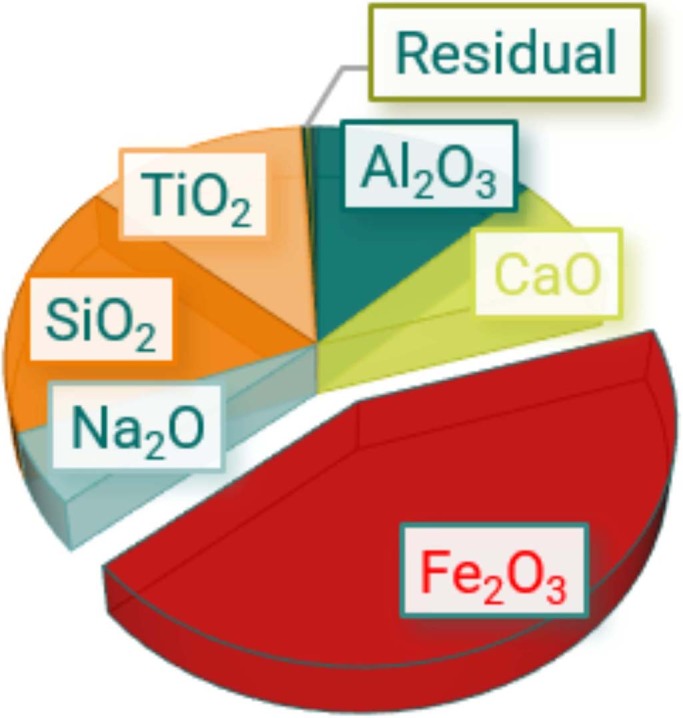
Graphical representation of the main oxides and their fractions within the investigated red mud. The complete oxide composition of the investigated red mud is 38.4 ± 2.08 wt% Fe_2_O_3_, 15.43 ± 0.44 wt% SiO_2_, 11.18 ± 3.31 wt% Al_2_O_3_, 10.96 ± 0.31 wt% TiO_2_, 6.52 ± 0.39 wt% CaO, 4.43 ± 0.24 wt% Na_2_O, 0.25 ± 0.01 wt% P_2_O_5_, 0.21 ± 0.00 wt% Cr_2_O_3_, 0.19 ± 0.05 wt% MgO and 0.16 ± 0.01 wt% ZrO_2_. The composition was determined with inductively coupled plasma optical emission spectroscopy performed on a Thermo Scientific iCAP 7000. All measurements were performed in triplet, from which the uncertainty was calculated as the standard deviation. Before the measurements, the samples were prepared with microwave digestion using HClO_4_, HNO_3_ and HF, followed by a subsequent complexation step with H_3_BO_3_.

**Extended Data Fig. 2:**
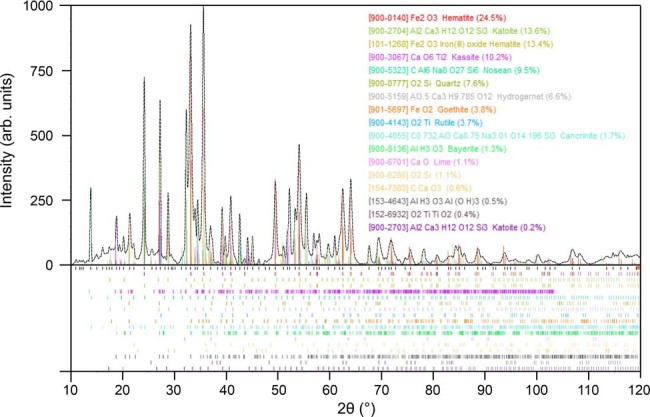
XRD data analysis of initial red mud sample with corresponding list of identified phases, both marked with peak markers and index list. For visualization purposes, the different peak markers are exaggerated or miniaturized for easier separation of the different peak indicators. The fraction of each phase is provided in the legend in wt%. The corresponding fraction of unidentified peaks is 3.6% of the total peak signals. The numbers in brackets refer to the COD ID of the corresponding phase, that is, the identification number in the Crystallography Open Database (COD), accessed 11 July 2022.

The chemical composition and crystallography aspects of the investigated red mud are documented in Extended Data Figs. 1 and 2 and Extended Data Tables 1 and 2. We show that the reduction pathway imposed by this simple processing transforms the solid red mud into a complex, viscous oxidic melt. The oxide liquid zones that are enriched in iron get preferentially reduced into a less viscous metallic liquid iron, which coalesces as nodules within the melt without reincorporating oxidic liquid portions (Fig. 1d). This means that iron (directly usable for subsequent steel production without any further refinement; see Fig. 1d) can be extracted from red mud in one single process step.

From a kinetics perspective, this process exploits both fast diffusivity of elements and the enhanced reactivity of a reducing plasma, whilst also using the density and mass difference between constituents to promote macroscopic mass partitioning that reliably enables iron separation from the remaining oxide portions. In the following, we show in detail how red mud can serve as a sustainable feedstock for green ironmaking. This creates a sustainability nexus between the two largest mass-produced metal groups, iron and aluminium. This approach can help to solve two of the most pressing environmental problems of our time, namely, use of red mud waste and production of iron without any direct CO_2_ emissions. We show the detailed thermodynamic and kinetic mechanisms and reaction pathways that drive the sequential cascade of the underlying redox reactions occurring during the reduction of red mud with hydrogen plasma (see also Supplementary information section ‘Thermodynamic calculations’). Our findings also provide insights on how to better exploit red mud as an economically viable and sustainable resource, rather than a costly environmental hazard.

## Reduction performance and Fe extraction

**Extended Data Fig. 3:**
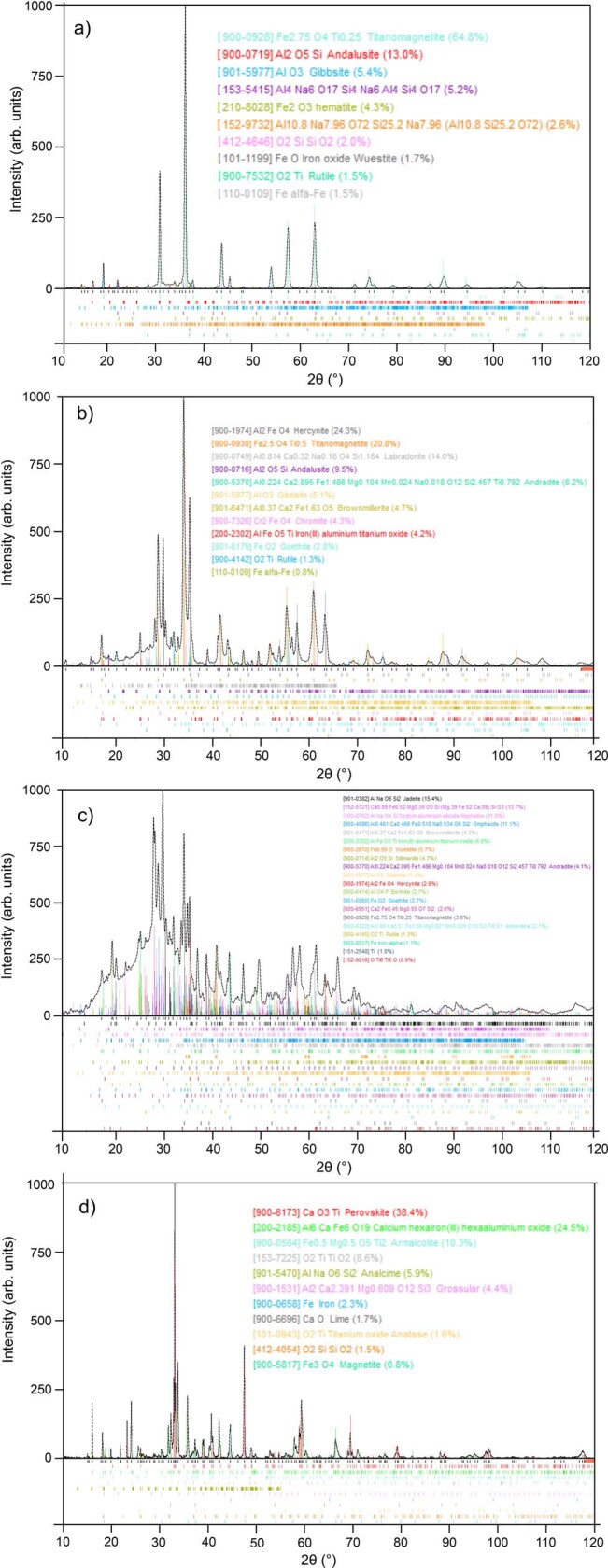
XRD data analysis. XRD data analysis of 1 min (**a**), 5 min (**b**), 10 min (**c**) and 15 min (**d**) reduced red mud samples with corresponding list of identified phases, both marked with peak markers and index list. For visualization purposes, the different peak markers are exaggerated or miniaturized for easier separation of the different peak indicators. The fraction of each phase is provided in the legend in wt%. The corresponding fraction of unidentified peaks is 1.45% 1.65%, 1.92% and 2.73% of the total peak signals for **a**, **b**, **c** and **d**, respectively. The numbers in brackets refer to the COD ID of the corresponding phase, that is, the identification number in the Crystallography Open Database (COD), accessed 11 July 2022.

Red mud was processed under a lean hydrogen plasma arc (Ar-10%H_2_) ignited at 200 A, as detailed in Methods. This reduction process results in the formation of pure iron that solidifies in the form of nodules within the residual oxide (see ‘reduced red mud’ specimen in Fig. 1d), an evidence of complete mass separation of the liquid portions, namely, the one enriched in oxygen and the other in iron. The remaining oxide domains solidify as a dark-obsidian-like structure with highly reflective cleavages, indicating a glassy structure of the remaining oxides. The metallic and oxide portions of each sample were mechanically separated. By weighing the individual sample portions and quantifying the corresponding phases with X-ray diffraction (XRD), the reduction kinetics of the red mud oxides into iron was traced and documented in Fig. 1e (see also the ‘Phase quantification’ section in Methods). The complete interpretation of the diffraction data is reported in Extended Data Fig. 3. The analytical protocol adopted here also permitted the evaluation of the oxygen removal and iron partitioning between the unreduced oxide portions and the metallic nodules (Fig. 1e). From the time evolution of these quantities, presented in the diagram in Fig. 1e, the increasing iron extraction with reduction time is visible (green curve).

Figure 1e also shows that the oxygen content in the oxide portions (blue line) slightly fluctuates around 35 wt% over the course of reduction, whereas the corresponding content of iron (red curve) remains nearly constant at 30 wt% during the first minute of the process. The figure also shows that the sample experiences a mass loss of about 3 g during the first minute (bar diagram in Fig. 1e), which corresponds to the thermal decomposition of haematite (Fe_2_O_3_) into magnetite-based (Fe_3_O_4_) species^11^, as well as the evaporation of clay-based oxides^12^ (Extended Data Figs. 1 and 2), together with a small degree of reduction to about 2 wt% of metallic iron. Afterwards, the predominant reduction of magnetite oxides leads to the increasing formation of metallic iron, thus decreasing the Fe content in the remaining oxide portion of the samples (red curve in Fig. 1e). About 30 wt% of the 10-min reduced sample is composed of iron (that is, 2.6 g of metallic iron was obtained after 10 min reduction and the total mass of the sample was 8.8 g). This number can also be translated into 62.4% of metallization when considering that the total amount of iron present in the initial 15 g of red mud was approximately 4.17 g. This value in fact constitutes up to 70% metallization when loss of ignition and evaporation effect are considered. Further discussion on this topic is provided in Supplementary information section ‘Evaporation effect and impact of high energy reduction’.

In other words, 2.6 g of metallic iron was extracted from 15 g of red mud, mostly in the form of large macroscopic nodules (see Fig. 1d). These nodules represent, on average, 98 wt% of the obtained metal, which is of high purity, as discussed further in Supplementary information section ‘Iron nodules purity’. The experimentally obtained amount of 2.6 g Fe is practically the same as that predicted through thermodynamic calculations for optimal extraction conditions (2.67 g Fe), which is documented in Supplementary information section ‘Theoretical limits of Fe extraction’. In that section, and as a complementary evaluation, we also investigated the impact of the basicity of the red mud on the theoretical Fe yield through hydrogen-based reduction. Both the experimentally observed Fe yield and the results reported in the ‘Theoretical limits of Fe extraction’ section allow us to infer that the reduction process conducted in this work proceeds near the thermodynamic equilibrium.

The reduction process by means of hydrogen plasma also brings about a neutralization effect of the reduced red mud. The residual oxides have a near-neutral pH value, which decreased from the original pH value of 10.5 of the red mud to a value of pH 9 after 5 min reduction and to pH 7.5 after 15 min reduction (Methods). As such, the process is not only valuable in extracting metallic Fe but also in rendering the residual material into a neutral product that can be potentially used directly in other industries (such as the construction industry) without costly neutralization processing.

## Chemistry evolution and phase transitions

**Fig. 2:**
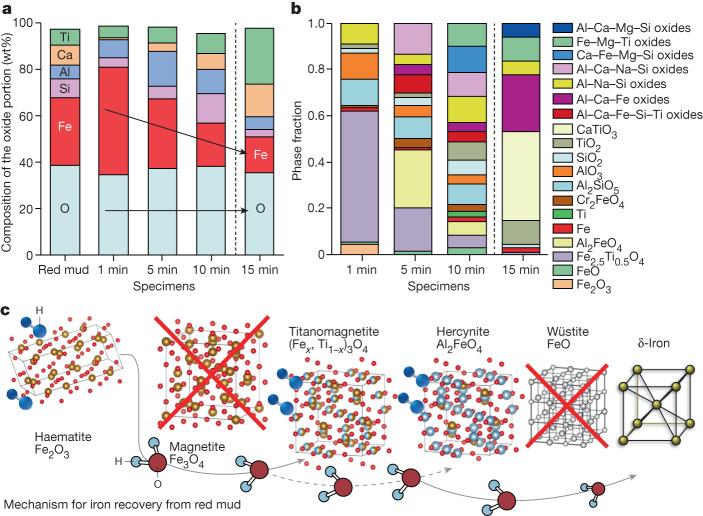
Phase evolution of red mud with hydrogen plasma processing and mechanism of iron recovery. **a**, Normalized weight fraction of the main chemical elements within the remaining oxide portions of the partially reduced red mud samples (1–15 min) in a lean hydrogen-containing plasma (Ar-10%H_2_). The black arrows indicate that the trends of iron and oxygen change with reduction time. **b**, Phase weight fraction in the remaining oxide portions of the partially reduced red mud samples, showing the differing complexity and evolution of oxides with reduction time. For clarity, the individual complex oxides with more than three metallic constituents are shown as individual groups. **c**, Chemical mechanism for iron recovery from red mud by means of hydrogen plasma reduction.

To explain the complete picture of the chemistry evolution over the reduction process and reveal the underlying mechanisms for iron extraction, the chemical composition of the constituents entrapped in the crystalline oxide portions of the partially reduced samples were analysed using XRD analysis and are presented in Fig. 2a. The data were normalized by the total weight of each reduced specimen (that is, summing the weight of both oxide and metallic portions) to avoid scattering effects from sample to sample owing to evaporation of volatile compounds, such as clay and water, during the first minute of the reduction. Figure 2a provides a clear insight into the linear decrease of the Fe content within the remaining oxides with reduction time (depicted by the black arrow). The oxygen content after 1 min reduction remains steady with reduction time, indicating the strong selectivity of our proposed method for extracting Fe from the oxide portion through two chemically driven mechanisms. First, through oxygen transfer from the iron oxides to the other oxides whose cations have higher affinity to oxygen than iron and, second, through oxygen removal by means of reaction with hydrogen plasma species from the arc, leading to water formation as a redox product of the hydrogen-based reduction of iron oxides (for example, Fe_3_O_4_ + 8H^+^ + 8e^−^ → 3Fe + 4H_2_O). More details on the thermodynamic mechanisms for the hydrogen-based reduction of molten red mud is provided in Supplementary information section ‘Thermodynamic calculations’.

The phase quantification of the oxide portions obtained using XRD also provides an important and complementary insight into the competing chemical side reactions during the reduction process. As shown in Fig. 2b, the complexity of the oxide fraction increases with reduction time, which also correlates well with the increased weight fraction of Ti, Al, Ca and Si within the oxide portion (see Fig. 2a). The reduction after 1 min shows the dominating presence of titanomagnetite (Fe_2.5_Ti_0.5_O_4_), which follows the thermal decomposition of haematite. Surprisingly, the formation of wüstite (FeO) is less prevalent, indicating that the formation of pure Fe occurs rapidly and directly from the titanomagnetite, rather than through intermediate formation of wüstite, as observed for the plasma-based reduction of pure haematite^15^. Such behaviour suggests an autocatalytic reaction also with the present oxides that allow such direct transitions to occur. With increasing reduction time, the titanomagnetite is reduced and with it the formation of hercynite (Al_2_FeO_4_) occurs because of the high presence of AlO_2_^−1^ species in molten red mud (Supplementary Fig. 1e). Throughout the following reduction, these two phases correspond to the main Fe carriers within the oxide portion of the samples. The mechanisms for iron formation are given in Fig. 2c. Figure 2b also shows that, after 10 min of reduction, Ti-enriched zones form, indicating that the reduction process can potentially be used to extract other metals besides Fe. When increasing the current to 800 A to ignite the arc (as discussed in Supplementary information section ‘Evaporation effect and impact of high energy reduction’) and the associated overexposure of the melt to the reducing plasma, shown by the 15 min reduction sample (Fig. 1e), the oxide shows a considerably simpler state, which is depleted of elements with lower weight and higher vapour pressure (Al and Si), but enriched with Ti and Ca that form thermally more stable oxides^16^. Despite the higher energy input, a small amount of Fe remains within the oxide portion, bound mostly within complex Ti-based and Ca-based oxides.

## Microstructure and chemical partitioning

**Fig. 3:**
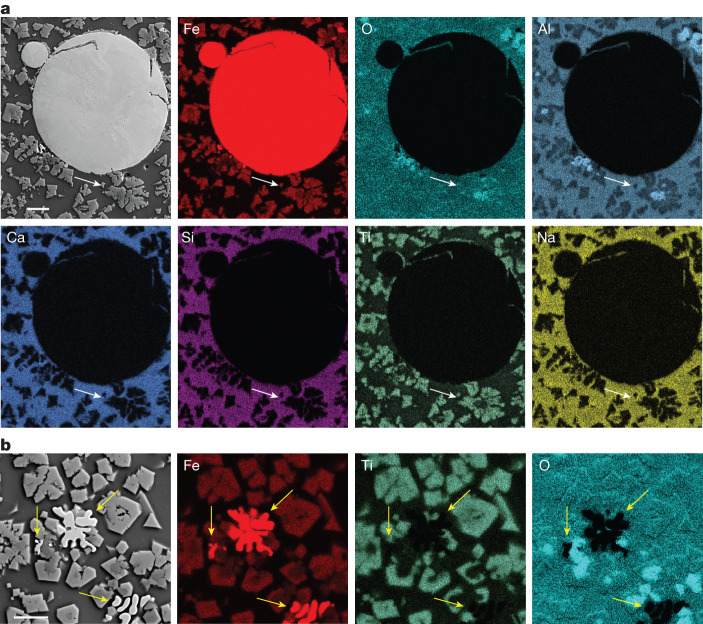
Microstructure of red mud after 1 min processing with hydrogen plasma. **a**, Microstructure of a red mud processed for 1 min under a lean hydrogen plasma (Ar-10%H_2_) and corresponding local chemical partitioning between fresh-formed iron droplets and the remaining oxide substructures, examined by means of EDX in a scanning electron microscope. The white arrows indicate the presence of an Al-rich phase hercynite (Al_2_FeO_4_), mostly accompanying the Fe-rich phase. **b**, Microstructure and local chemical evidence for the formation of pure iron in a splatter-like morphology (arrowed) from titanomagnetite (Fe_2.5_Ti_0.5_O_4_). Scale bars, 20 μm.

To understand the phase evolution and local chemical partitioning during the reduction process, systematic cross-section analysis with scanning electron microscopy (SEM) was performed on the reduced and solidified samples. In Fig. 3a, the microstructure of the red mud reduced for 1 min is presented. The corresponding local chemical partitioning among the constituents of this specimen was examined with energy-dispersive X-ray spectroscopy (EDX). The microstructure investigation confirms that, already, 1 min of exposure to hydrogen plasma enables the formation of micron-sized iron domains, which solidify in the form of spherical nodules and remain entrapped within a predominantly oxide matrix composed of Al, Si, Ca and Na. Oxides in the form of dendrites also populate this microstructure, whose chemistry can be associated with that of titanomagnetite, as previously revealed by the XRD results shown in Fig. 2b. Also, a few Al-enriched oxides form within the titanomagnetite, suggesting the initial formation of hercynite (arrowed in Fig. 3a), in agreement with the diffraction data shown in Fig. 2b. In specific regions, the initial formation of Fe with a splatter-like morphology can be seen, as indicated by the yellow arrows in Fig. 3b, indicating the direct transformation of titanomagnetite into pure Fe, which confirms the mechanism proposed in Fig. 2c.

**Fig. 4:**
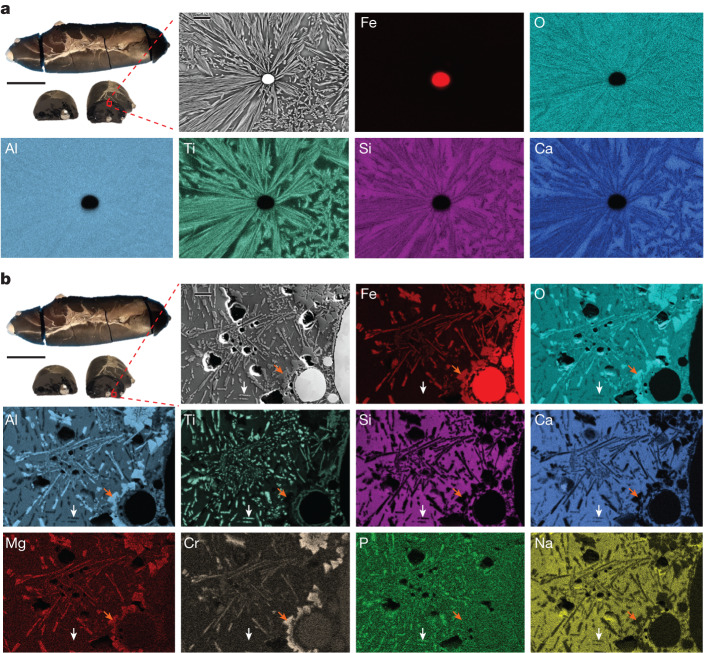
Microstructure of red mud after 10 min of processing with hydrogen plasma. Microstructural and local chemical composition of the sample partially reduced for 10 min under hydrogen plasma (Ar-10%H_2_), examined by means of EDX in a scanning electron microscope. **a**, This region is about 1 mm below the top surface of the sample, showing a highly concentrated arrangement of oxides enriched with Al, Ca, Si and Ti and depleted of Fe. Scale bars, 1 cm (main), 10 μm (inset). **b**, Region examined close to the bottom of the specimen, depicting the complex resulting microstructure after reduction, showing the individual oxides and the metallic portions. The orange arrows show the chunky oxide domains enriched in Al, Fe, Mg and Cr. The white arrows show the constituents enriched in Ti that, in some cases, coincide with P enrichments. Scale bars, 1 cm (main), 20 μm (inset).

The microstructure of the 10 min reduced sample, presented in Fig. 4, shows a much more spatially dynamic system as previously inferred from the XRD analysis. Unlike the 1 min sample, the 10 min specimen shows a considerable chemical and microstructural difference between its top and bottom portions. The microstructural and local chemical characterization, conducted in a region approximately 1 mm below the top surface of the solidified sample, reveals a strong depletion of Fe and high enrichment of Ti, Si, Ca and Al oxides. The example microstructure provided in Fig. 4a shows an isolated 5-μm-sized Fe droplet surrounded by titanium-enriched oxide dendrites. Figure 4a suggests that Fe droplets are scarcely found in the top regions of the sample and also chemically detached from other elements within the oxide melt. Furthermore, the top oxides confirm the subsequent formation of complex oxides at high temperatures and the successive Ti purification from the other elements found in the oxide portion.

By contrast, the microstructural features lying in the bottom of the same sample are rather distinct (Fig. 4b). The region investigated was examined adjacent to a large Fe-rich domain, to reveal the distinct chemical partitioning among the different phases. Figure 4b shows chunky domains composed of Al, Fe, Mg and Cr oxides, as indicated by the orange arrows. These domains show a separation based on local chemistry. Although Al and Fe are evenly distributed across the entire domains, Mg and Cr accumulate along their peripheral regions. These features show the complexity of the oxide transitions through different phases, enriched with several constituents, which result in the binding of the remaining Fe to the oxide portion, yet revealing opportunities for further Fe extraction from such domains. Furthermore, the microstructure also shows Ti-rich elongated regions, marked by white arrows. They also show high depletion of Ca, Si, Al and Na, as well as O, suggesting that these phases consist mostly of Ti, as also inferred from the XRD analysis (Fig. 2b). Some of the Ti-enriched zones also contain P, which probably forms as a result of the preferential formation of coordinated structures of transition-metal phosphates with Ti in the liquid state^17^. The residual glassy matrix of this sample, present between the different phases, has a chemical composition similar to that of the sample reduced for 1 min.

## Effectiveness of hydrogen plasma

**Fig. 5:**
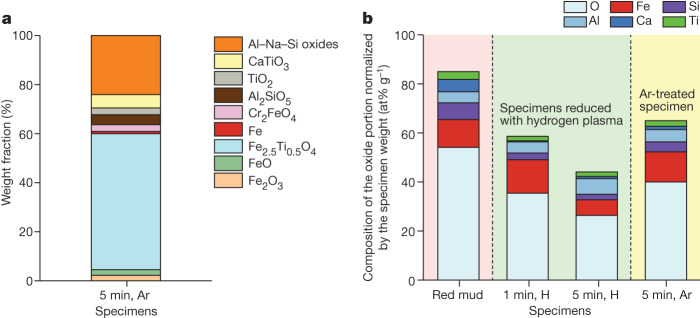
Comparing the effect of non-reducing and reducing plasma gas species on red mud processing. **a**, Phase weight fraction in the remaining oxide portions of the partially reduced red mud sample after 5 min reduction in pure inert Ar plasma, an experiment serving as a reference scenario with non-reducing atmosphere. **b**, Chemical composition of the oxide portions of the processed red mud with reducing hydrogen (for 1 min and 5 min) and inert argon (5 min) plasmas in comparison with the corresponding chemical composition of the original red mud. The composition was normalized by the mass of each corresponding specimen to visualize the weight loss of the oxide portion with the reduction process.

**Extended Data Fig. 4:**
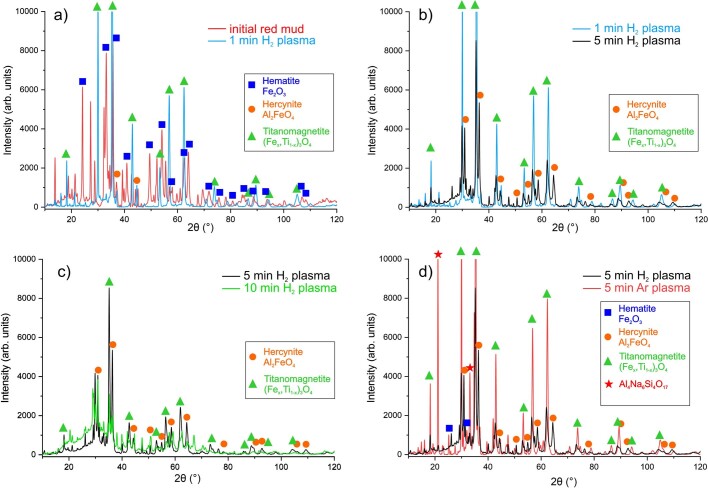
Diffraction data for red mud samples processed under hydrogen-containing and inert Ar plasmas. Comparative illustration of the XRD pattern of the initial state of the red mud against the oxide portion of the 1 min reduced red mud sample under hydrogen plasma (**a**), the oxide portion of the 1 min reduced red mud sample against the oxide portion of the 5 min reduced red mud sample (**b**), both under hydrogen plasma, the oxide portion of the 5 min reduced red mud sample against the oxide portion of the 10 min reduced red mud sample, both under hydrogen plasma (**c**), and the oxide portion of the 5 min reduced red mud sample under hydrogen plasma against the oxide portion of the 5 min reduced red mud sample under Ar plasma (**d**). The peaks of the main iron-holding phases are correspondingly marked with designated symbols. For the example shown in **d**, the additional strong oxide phase without Fe is also marked with the red star.

So far, the successful reduction of red mud with a 10% hydrogen-containing plasma is confirmed through a simple and fast process in which the reduction proceeds simultaneously through atomic-scale chemical partitioning and viscosity-driven macroseparation between metals and the lighter oxides. However, the actual effect of the thermal decomposition of the oxides and exposure to the high-energy-carrying plasma in correlation with the used hydrogen gas is not decoupled. To gain insight into this, further experiments were carried out by processing 15 g of red mud under pure (non-reducing) Ar plasma for 5 min and using the same apparatus as before, as a reference process. The processed sample was powdered and examined using XRD and the obtained results are shown in Fig. 5a. The sample obtained contains predominantly titanomagnetite, which is associated with the thermal decomposition of haematite (see Extended Data Fig. 4 for comparative relation of XRD peaks). Surprisingly, despite the lack of reducing agents within the inert Ar plasma, the sample shows minor amounts of both FeO and pure Fe. However, the content of Fe produced by such an inert plasma is considerably lower (the total amount is only 0.27 g, about 7% of the total available Fe) compared with the amount obtained after 5 min reduction with reducing hydrogen plasma (1.34 g of pure Fe, about 33% of the total available Fe), for the same amount of 15 g input material. The formation of the minor Fe-rich zones without hydrogen can be explained by the evaporation of minor quantities of oxygen from the melt followed by the subsequent internal exchange of the remaining free oxygen. This reaction sequence leads to the formation of other oxide species such as SiO_2_ and Ti_2_O_3_ that have higher oxygen affinity and permits the precipitation of minor metallic Fe fractions, as also supported by the thermodynamic calculations documented in Supplementary Fig. 1.

Furthermore, under the inert Ar plasma process, the residual oxide states hold considerable amounts of clay-based oxides (Fig. 5a) and water, giving a reason for the lower mass loss with Ar process time (30.1% mass loss) when compared with the counterpart sample processed with hydrogen plasma (40.9% mass loss). Finally, through the extracted chemistry of the oxide portion of the Ar-processed sample (Fig. 5b) and subsequent comparison with the chemistry of the samples treated with reducing hydrogen plasma, the much stronger effect of hydrogen gas and ionic species is demonstrated. The Ar-treated sample shows higher O content in the oxide, as well as only very small changes in the Fe content, compared with its hydrogen plasma counterpart. Moreover, the sample has higher O content than the hydrogen-reduced sample after 1 min, further supporting the sluggish O removal through evaporation and limited conversion of all oxides, which is also supported by the thermodynamic calculations (see Supplementary Fig. 1).

## Discussion

Our results provide direct evidence of the fast kinetics and preferential reduction of the haematite portion of red mud into pure iron. The effectiveness of the process is high, reaching 70% metallization after 10 min reduction. Furthermore, Fe loss through evaporation after 10 min reduction time is only about 7%. In comparison with other comparable schemes of Fe extraction from red mud^3^, our process results in high-purity Fe products with, on average, 95 wt% Fe content and negligible presence of detrimental elements, such as S, P and C, allowing for its direct use in steelmaking. The underlying chemical mechanism is considered to occur from the cascading effect of oxide transition from titanomagnetite to hercynite and then directly to pure Fe. The effect of microalloying of the magnetite-like structures with Ti and Si allows a more stable state of the magnetite type, which allows a more straightforward nucleation of Fe, without a pronounced formation of the intermediate wüstite (FeO), which is a diffusional limiting barrier in iron oxide reduction in both the solid^18^ and the liquid states^19^. By comparing a pure haematite sample with similar haematite weight as in the red mud sample, reduced with similar hydrogen plasma conditions^15^, the red mud is about 20% faster in metallization. This comparison shows a clear modification of the reduction capability of the molten haematite, which can be related not only to the possible catalytic effects of the extra gangue oxides (a thermodynamics-driven aspect)^20^ but also to their viscosity effect of the melt (a kinetics-associated phenomenon) and local fluid dynamics that are more deeply discussed in Supplementary information section ‘Fluid dynamics and implications on upscaling’.

As well as pure Fe extraction, the presented hydrogen-plasma-based reduction process of red mud also seems to be a feasible method to extract other metals, such as Ti, confirmed by the discovery of micrometre-sized Ti-enriched domains (Figs. 2b and 4b). This provides not only a reasonable process for treating mining waste material and conducting Fe extraction but also a feasible method to extract a variety of other precious metals in a single processing step. This suggests that the process can be expanded and further valorized to include extraction of various rare-earth metals such as Sc and Y, which can be obtained through subsequent processing of the remaining oxide-enriched portions with well-established extraction techniques^21–23^. This is especially suitable for red mud material originating from karst bauxite that is predominantly richer in rare-earth and precious elements^3^. Owing to the prior removal of Fe from the red mud by means of hydrogen-plasma-based reduction, the extraction of rare-earth metals, retained and enriched in the processed red mud, could be potentially more efficient than their removal from the unprocessed red mud because the presence of Fe and Fe-rich phases seem to be the main cause of the low efficiency of rare-earth-element extraction through conventional extraction techniques^23^.

From an economical point of view, the process might open financially viable avenues for industry, as the reduction processing is very fast and economic for most red mud compositions, as more deeply discussed in Supplementary information section ‘Technoeconomic assessment’. Despite the high energy input for the small material weight processed in this study, the process can be considerably lower in energy cost, with upscaling owing to the exothermic charter of the reduction process, catapulted by the highly reactive hydrogen radicals in the plasma, and the strong enhancement in reduction efficiency with increasing mass, as proved with pure haematite reduction^15^. From the perspective of the final product, the metallic Fe is directly usable for steel production as direct Fe charge. The purified residual oxide portions with their glassy-like state, mostly composed of highly thermally stable oxides and neutral pH, are ideal materials to be directly used in concrete, asphalt, paints and other construction binding materials^5,6^. With the detailed financial evaluation documented in the ‘Technoeconomic assessment’ section, we demonstrate that, even under challenging cost conditions, our plasma-based process can be financially viable for Fe-rich red mud waste, surpassing the conventionally based processing of iron ores for steel production, owing to the combined effects of less CO_2_ emissions and lower processing costs^24^.

In comparison with other treatment procedures of red mud for metal extraction, this process is the first of its kind to provide direct pure Fe extraction in a single clean processing step. It does not involve any type of pre-treatments or post-treatments, carbon as heat source or reductant, mineral roasting^8,14^ or acid/leaching treatments^3,25,26^, yet, it yields high metallization and neutralization of the final products. Furthermore, other EAF-based red mud processing procedures^8,14,24,27^ and roasting/pyrometallurgical schemes^3,8,25,26^ are carbon-based, making them environmentally problematic, thus only shifting the environmental impact associated with red mud valorization to other harmful (carbon-based) scenarios, rather than actually solving it. All in all, the proposed process results in highly efficient metal extraction and complete processing of red mud into valuable metals and nearly inert oxide-based sub-products, making it an environmentally friendly, economically attractive and sustainable method for long-term red mud waste management and use.

## Methods

### Material

The material used in this research is a dry red mud whose chemical composition and phase content are provided in Extended Data Figs. 1 and 2 and Extended Data Tables 1 and 2, respectively.

### Reduction experiments

Red mud samples with an average weight of 15 g were placed on the water-cooled copper hearth of a conventional arc melting furnace equipped with a tungsten electrode (6 mm in diameter). The furnace chamber (18 l) was flooded with a gas mixture of Ar-10%H_2_ at a total pressure of 900 mbar. An arc plasma was ignited between the electrode and the input material at 200 A. Simultaneous melting and reduction was conducted during 1 min of exposure to reducing plasma. After this, the arc was switched off, the sample solidified and the chamber atmosphere replenished with a fresh gas mixture of Ar-10%H_2_. This procedure was repeated a further five and ten times to produce samples reduced for 5 and 10 min, respectively. The same protocol was adopted to reduce a 15-g red mud sample under 15 min of exposure to reducing plasma, ignited at 800 A. A further sample was reduced for 5 min under an inert atmosphere of argon (total pressure of 900 mbar). The arc was generated using 200 A and the furnace chamber was replenished with a fresh argon atmosphere after the completion of every 1 min melting/reduction/solidification cycle. For each exposure time, at least three samples were produced and analysed.

### Phase quantification

Small pieces were cut from the solidified samples for microstructural analysis. The remaining portion was hammered to separate millimetre-sized iron portions from the remaining oxides. The corresponding oxide portions were further powdered and examined through XRD measurements. XRD was performed with a Bragg–Brentano configuration with a Cu Kα source. The 2*θ* scan range was set from 10° to 120° with a sampling step of 0.01°. For the local analysis of the Fe nodules, a microarray XRD set-up is used with the same parameters and an aperture slit of 0.5 × 0.5 mm^2^. The XRD data were analysed with a combination of Rietveld refinement^29^ and the Toraya method^30^. All XRD data were evaluated with initial separation of the crystalline peaks and amorphous background (if present), and the crystalline part was evaluated. Afterwards, the extracted amorphous part was also analysed through association of its form and small peaks formation to possible nominal crystalline peaks, which were then parametrically broadened to simulate the amorphous structure formation. A uniform lattice dilation was assumed for this purpose. The elemental chemical extraction for each sample was performed through the assumption of homogeneous phases with nominal compositions. The assumption is generally valid because of the melted state of the material at high temperatures under plasma and fast cooling of the sample to room temperature by means of the water-cooled Cu crucible that prohibits large mass transfer during sample solidification.

XRD also allowed for quantifying the micron-sized Fe domains that remained entrapped inside the oxide portions. The absolute masses of iron (that is, summing the millimetre-sized and micron-sized domains) obtained after 1, 5 and 10 min of reduction were 0.10, 1.34 and 2.60 g, respectively.

### Microstructural characterization

Representative samples were metallographically prepared by grinding and polishing and finalized with silica particle OPS polishing. The metallographic investigation of the samples was performed with a Zeiss Merlin microscope through high-resolution SEM, including EDX.

### pH evaluation

Individually processed samples (for more details, see the ‘Reduction experiments’ section) and samples of initial red mud material were powdered and dissolved in distilled water with pH 6.0 ± 0.2. The dissolvement process was performed with the introduction of 1 g of sample into 20 ml of distilled water at room temperature. The mixing was performed with magnetic stirring for at least 30 min and afterwards left to settle any remaining undissolved larger particulates. The pH testing was performed with different indicator papers: universal (pH range 1–14), comparator A (pH range 8–10), comparator B (pH range 9–13) and comparator C (pH range 6.2–7.8). Each sample solution was then measured with universal and at least one comparator pH paper based on the result of the universal test. The measurements were performed three times for the same solution, with intermediate mixing between each measurement. For each sample, at least two solutions were tested. The measurement deviation for each sample was within the 0.2 pH range.

### Thermodynamic calculations

Equilibrium calculations were performed using the software Thermo-Calc coupled with the TCS Metal Oxide Solutions Database (TCOX10) for the description of the liquid phases and the SSUB5 SGTE (Scientific Group Thermodata Europe) Substances Database for the description of the gas phase, which includes metallic and oxide vapours. For this purpose, a 15-g red mud mass with the chemical composition shown in Extended Data Table 2 was set as the boundary condition. The molten red mud was kept at 1,850 °C while simultaneously adding increasing quantities of a gas mixture of Ar-10%H_2_. Element partitioning was permitted among all constituents during the simulations. The total pressure was 1 × 10^5^ Pa. The obtained results are shown in Supplementary Fig. 1, which shows the amounts of phases and their corresponding chemical compositions as a function of the quantities of hydrogen inserted in the system.

### Technoeconomic assessment

The technoeconomic evaluations are performed with extracted values from refs. ^31–38^. The detailed description of the model, calculations and considered parameters are fully provided in Supplementary information sections ‘Technoeconomic assessment’ and ‘Technoeconomic assessment calculations’.

## Online content

Any methods, additional references, Nature Portfolio reporting summaries, source data, extended data, supplementary information, acknowledgements, peer review information; details of author contributions and competing interests; and statements of data and code availability are available at 10.1038/s41586-023-06901-z.

### Supplementary information

Supplementary Information

## Figures and Tables

**Extended Data Table 1:**
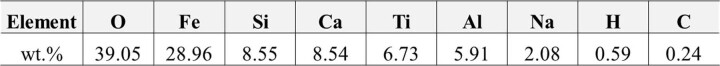
Approximate elemental composition of the red mud

Composition extracted on the basis of the ideal stoichiometry (set by the selected index cards) and fraction of the determined phases with XRD.

**Extended Data Table 2:**
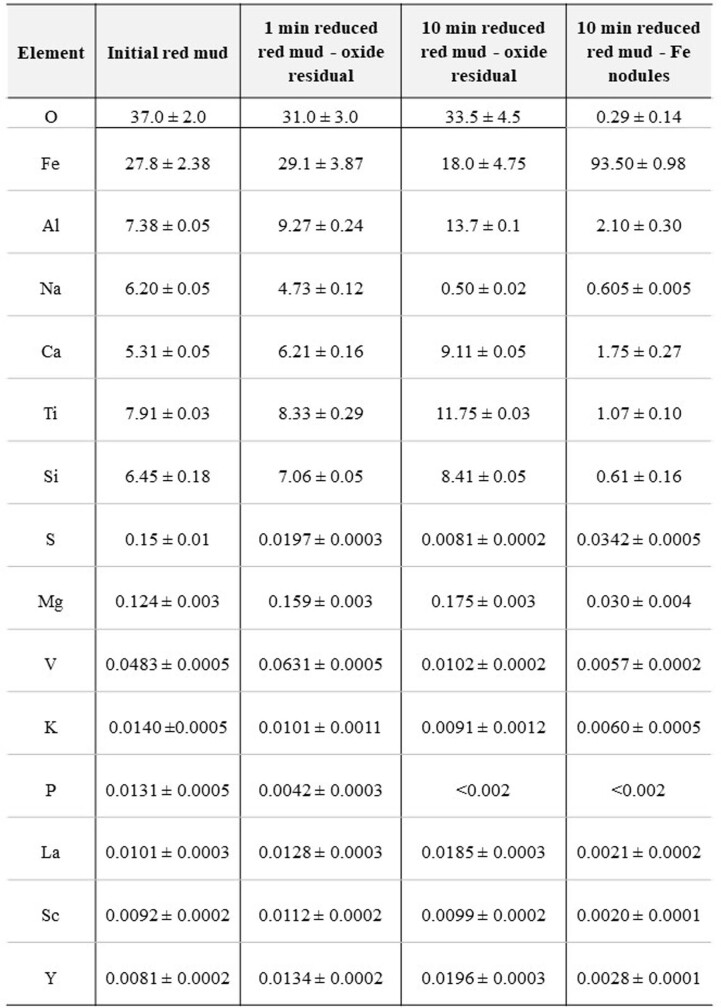
Chemical composition of processed red mud samples

Composition determined with inductively coupled plasma optical emission spectroscopy and X-ray fluorescence of the initial red mud oxide residue after 1 min and 10 min reduction time and of the Fe nodules after 10 min reduction time. The deviations include the sample-to-sample variation as well as the standard error of the measurement method. We performed X-ray fluorescence analysis following the standard DIN 51001:2003 RFA. All values are presented in wt%.

## Data Availability

Data are available from the corresponding author.
