# Supplementary material for: Green steel from red mud through climate-neutral hydrogen plasma reduction
Source: Nature. 2024 Jan 24;625(7996):703–9. doi: 10.1038/s41586-023-06901-z (PMC10808056; doi:10.1038/s41586-023-06901-z)
Supplement: Supplementary file 1 — Supplementary Information [file 41586_2023_6901_MOESM1_ESM.pdf]

nature portfolio

https://doi.org/10.1038/s41586-023-06901-z

Supplementary information

Green steel from red mud through climate-neutral hydrogen plasma reduction

In the format provided by the authors and unedited

Supplementary material

Green steel from red mud through climate-neutral hydrogen plasma reduction

Matic Jovičević-Klug[1]†, Isnaldi R. Souza Filho[1]†*, Hauke Springer[1,2], Christian Adam[3], Dierk Raabe[1]

[1] *Max-Planck-Institut für Eisenforschung; 40237 Düsseldorf, Germany.*
[2] *Institut für Bildsame Formgebung, RWTH Aachen University; 52072 Aachen, Germany.*
[3] *Bundesanstalt für Materialforschung und -prüfung; 12205 Berlin, Germany.*

†these authors contributed equally and figure together as first authors.

* corresponding author: i.souza@mpie.de

Geographic dependency of red mud and current state on utilising red mud for Fe extraction

Geographically, red mud is predominantly concentrated in countries with a high volume of bauxite and alumina production, namely Australia, China, Guinea, Brazil and India[40], which contribute about 80 % of the amount of fresh red mud accretion. Specifically, the concentrated production in China yields a red mud accumulation up to as much as 88 million tons per year[40]. The growing environmental concern consequentially led to an increased interest in the utilisation of red mud to avoid the issues of its disposal[13]. Depending on its origin in terms of ore deposit and refining technology, red mud can hold varying amounts of hematite, from as low as 7 wt.% (e.g., in China[3]) up to 60 wt.% (e.g., in Australia[14]). However, on average the hematite values range between 30 and 45 wt.% for most of the red mud deposits[3,13,14]. Since the weight fraction of iron oxides in red mud can be as high as 60 wt.%, opportunities for sustainable iron recovery from this residual material is a particularly worthy topic in the context of red mud recycling and reutilisation. Only few attempts have yet been made to turn red mud into a valuable and sustainable feedstock and to recover iron and other metals from it, as they are bound in complex and thermodynamically stable mineral phases[8,41]. This suggests that efficient metal extraction from red mud requires smelting-based reduction processes, in which solid compounds are first melted allowing for the

transformation of their complex mineral structures into less complex ionic variants, including free ionic oxygen (O^{-2}).

Adopting this concept, pig iron, a Fe~4.5wt.%C precursor compound for steelmaking, was successfully produced by processing red mud as a feedstock together with coke as reductant through carbothermic smelting processes either in a blast furnace[29,41] or assisted by argon plasma treatments[25]. However, due to the use of coke, a reducing agent which produces massive CO_2 emissions, these approaches thus simply shift the pollution problem from one industry sector to another. Also, coke-based processing of oxides yields raw iron containing excessive amounts of C, S and P (due to partitioning from the coke to the metal), which typically increases the required efforts in costly secondary metallurgy treatments before it can be used for final steel production.

To avoid CO_2 emission during processing of red mud, hydrometallurgical routes have been developed. In these approaches, leaching is utilised for extraction of iron and other elements[3,13,14]. These processes allow to extract high amounts of the targeted material, but in the form of dissolved ions in solutions, which requires further post-processing, such as solvent extraction. As a result, this may render the process uneconomical for large-scale extraction of more abundant elements such as iron[3]. Additionally, these processes are not entirely sustainable due to the utilisation of acids that result in CO_2 generation during preparation. Furthermore, after their utilisation, the acids and leaching solutions commonly require neutralisation, a practice that can introduce additional pollution and associated carbon footprint even further.

Thermodynamic calculations

The reduction of oxides contained in red mud is governed by thermodynamics, i.e., it is determined by the free energy of formation of the different oxides in dependence of the temperature[42]. The preferential hydrogen plasma-based reduction of the iron oxides to metallic iron is governed by the direct exchange of the oxygen from the molten FeO towards the formation of water (e.g., $FeO + 2\,H^+ + 2\,e^- \rightarrow Fe + H_2O$). This is due to the fact that iron exhibits the lowest affinity to oxygen of the metals within in the red mud[42]. The oxygen that remains in the liquid preferably bonds with the other metals to form non-iron based ionic structures (SiO_4^{-4}, Ti_2O_3, AlO^-), as confirmed by the thermodynamic calculations shown in Fig. S1e. Thus, the resulting oxygen exchange among the different ionic species in the liquid and its removal with hydrogen lead to the

formation of metallic liquid iron and an oxide melt which are immiscible liquids, as confirmed by the thermodynamic calculations documented in Fig. S3a-c.

The thermodynamic calculations in Fig. S1e-f show that the ionic variants in the oxide liquid display a varying affinity to oxygen, and that the Fe-oxide ionic species have the tendency to release it as free oxygen in the presence of hydrogen gas in comparison to other oxide species such as SiO_4, TiO_2, CaO and NaO. As a result, the Fe ions preferentially dissociate from the oxygen ions to form a metallic liquid. To this extent, the individual metallic ions show that only Fe, Cr and Na are removed from the oxide through reduction and liquid phase separation or evaporation (see Fig. S1b,d,f). The free oxygen released from Fe-oxide species seems also to be partially incorporated into the SiO_4^{-4} species that recombine into the SiO_2 species (seen by the increase of SiO_2 fraction in Fig. S1e). Later the SiO_2 species show a decline in their site fraction with the progress of the reduction, which is directly correlated to the presence of Si in the gas phase, as shown in Fig. S1d. This shows that - unlike Fe, Cr and Na - Si preferentially evaporates as oxygen-carrying structures (e.g., SiO) rather than as a metallic ion species. In contrast, Ca, Al and Ti do not show a predominant evaporation with the reduction path. However, Ti has a tendency of changing its ionic character from TiO_2 to Ti_2O_3 with progressing reduction, a result that suggests its partial reduction with hydrogen plasma.

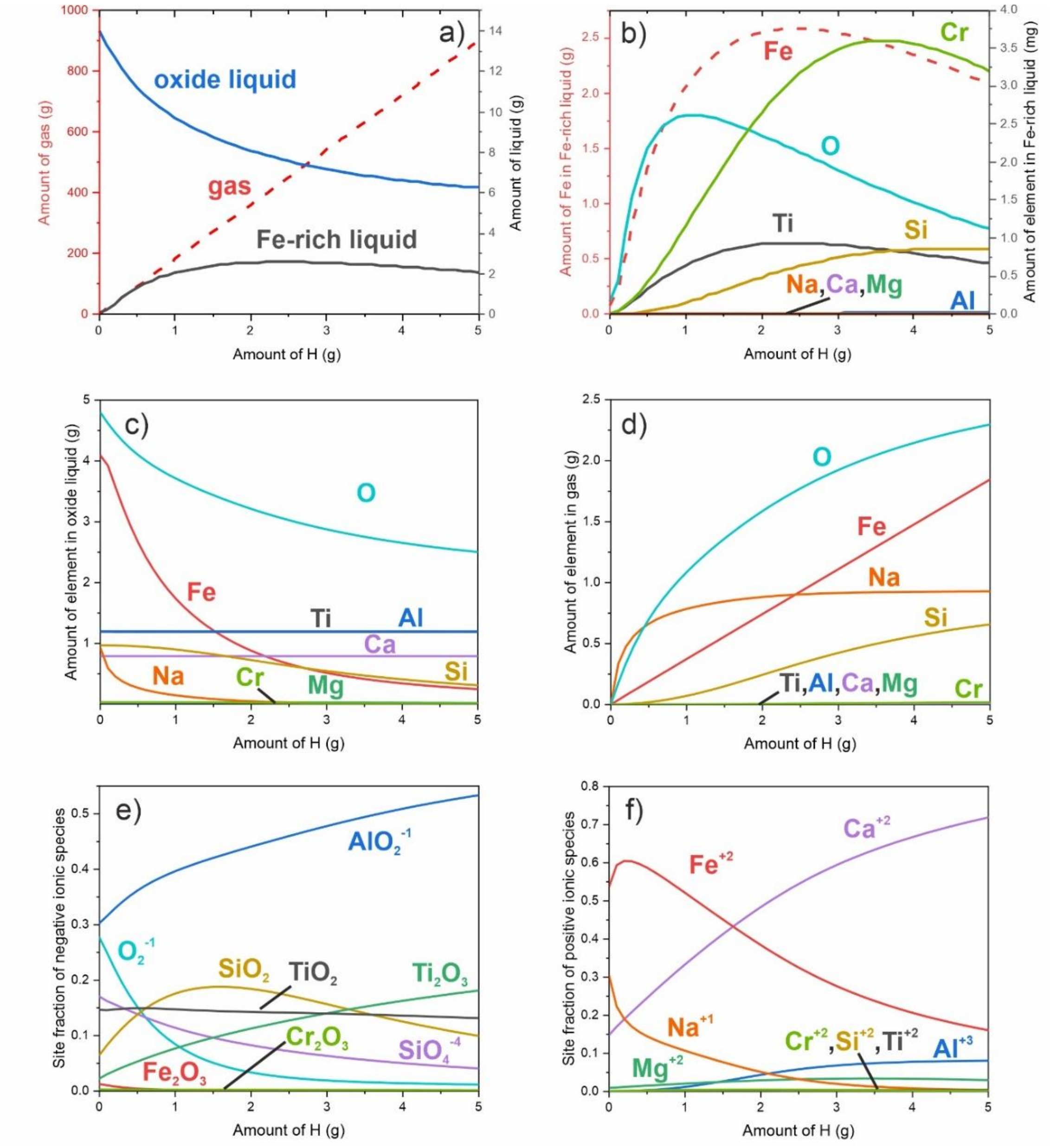

Fig. S1: **Results of thermodynamic calculations of the red mud exposed to Ar-10%H2 gas at a temperature above its melting point.** For the calculations a temperature of 1850 °C was chosen based on the amount of the individual phases and material losses that were determined from the experiments. The initial weight of the sample is considered as a 15 g red mud material with the initial weight loss due to evaporation of volatile species and water. The chemical composition of the red mud from Extended Data Table 2 was used for the calculations. To simulate the progress of the reduction, the hydrogen gas in atomic state is incrementally increased (from 0 to 5 g) in the gas system. **(a)** Amount of the phases existing at reduction temperature. **(b)** Number of individual elements (in g) within the Fe-rich liquid phase. **(c)** Number of individual elements (in g) within the oxide liquid phase. **(d)** Number of individual elements (in g) within the gas phase that correlates to the evaporation of the individual elements (except O, which reacts with H to form water). **(e)** Site fraction of negative ionic species within the oxide liquid. **(f)** Site fraction of positive ionic species within the oxide liquid.

Evaporation effect and impact of high energy reduction

Small quantities of some Fe-bearing volatile constituents can evaporate during to the ignition of the arc (the loss of ignition, LOI, of our experiments is 6.4 wt.%, see section "Total hydrogen consumption" in this supplementary material). Thus, the total metallisation can be related to the total available iron in both, the remaining oxide portions and the extracted Fe nodules, i.e., not considering the iron that inevitably gets evaporated together with the volatile compounds containing it. Based on these considerations the actual metallisation rises to 70 %. Within our study, we emphasise that the metallisation corresponds exclusively to the acquired bulk metal that is directly usable as a feedstock and does not require any further post-processing such as filtering, precipitation, dehydration, pelletizing or other purification processes that are required in other extraction methods[3,13,14].

To showcase the effects of the extreme reduction conditions of the process, 15 g of red mud was overexposed (15 min) to the lean hydrogen plasma ignited at a higher current of 800 A, and not 200 A as conducted before. Particularly, for this experiment, the total mass loss of the sample is considerably higher (see the bar diagram in Fig. 1e) due to the excessive thermal evaporation of the material, a fact that also leads to losses of pure Fe domains thus dropping the metallisation degree to 43 %. The effect of the evaporation can be also assessed via the thermodynamic calculations shown in Fig. S1d. This figure predicts the highest evaporation of Fe followed by Na and Si with continuous exposure to hydrogen-containing atmospheres, whereas the other elements display a negligible evaporation tendency. The tendencies of the evaporation with hydrogen plasma reduction indicate that a balance between reduction time and parameters needs to be established in order to obtain the maximum amount of metallic iron while minimising evaporation losses. Nevertheless, excessive metallic evaporation in large scale processing can be further minimised with proper slag development, which can limit the evaporation losses of iron up to 2.5 wt.% in standard steelmaking with EAF[46].

Iron nodules purity

Regardless of the reduction time and the applied current, the resulting metallic nodules are of high iron yield, displaying a composition of 99.1 wt.% Fe on a local scale (see Table S1 and Fig. S2) as determined with energy-dispersive X-ray spectroscopy (EDX). Due to the presence of Cr

oxides in red mud, up to 0.43 wt.% of Cr is also found dissolved in the iron nodules. When also considering Cr, the total metallic content of the iron nodules reaches up to 99.6 wt.%, which falls within industrial standards of technically pure iron alloy[43]. The high purity and homogeneity are also confirmed with EDX mapping of the larger iron nodules (see Fig. S3a). The electron backscatter diffraction (EBSD) maps shown in Fig. S3a,c-e as well as the XRD data presented in Fig. S3f, show that the iron nodules are purely BCC α-iron with large grain sizes in the order of 100 µm, and devoid of other phases within the nodules (see Fig. S3a-f). However, some of the nodules hold a minor amount of entrapped oxides, which can lower their purity down to 98 wt.% Fe at a local scale (Fig. S2) or to 92.5 wt.% at bulk scale (Extended Data Table 2). An example of a scanning electron microscopy (SEM) image, depicting the trapped oxide within an iron nodule is provided in Fig. S2. The entrapment of such minor oxide domains inside the iron is mainly due to the high solidification rates imposed by the water-cooled Cu hearth on which the red mud samples were processed in our experiments. However, it should be noted that the contamination of the iron with oxides can be likely reduced in large scale production in which the volume of processed material remains in liquid state for more prolonged times after switching off the arc, thus permitting lower cooling and better density induced separation.

Table S1: Chemical composition of iron sections (not including the regions displaying entrapped oxide material) from the extracted iron nodules determined with point EDX. The deviations include the sample-to-sample variation as well as the standard error of the measurement method. The values are presented in wt.%.

Element	Fe	Cr	Si	Ca	Ti
**wt.%**	99.12 ± 0.32	0.43 ± 0.04	0.29 ± 0.16	0.06 ± 0.05	0.10 ± 0.06

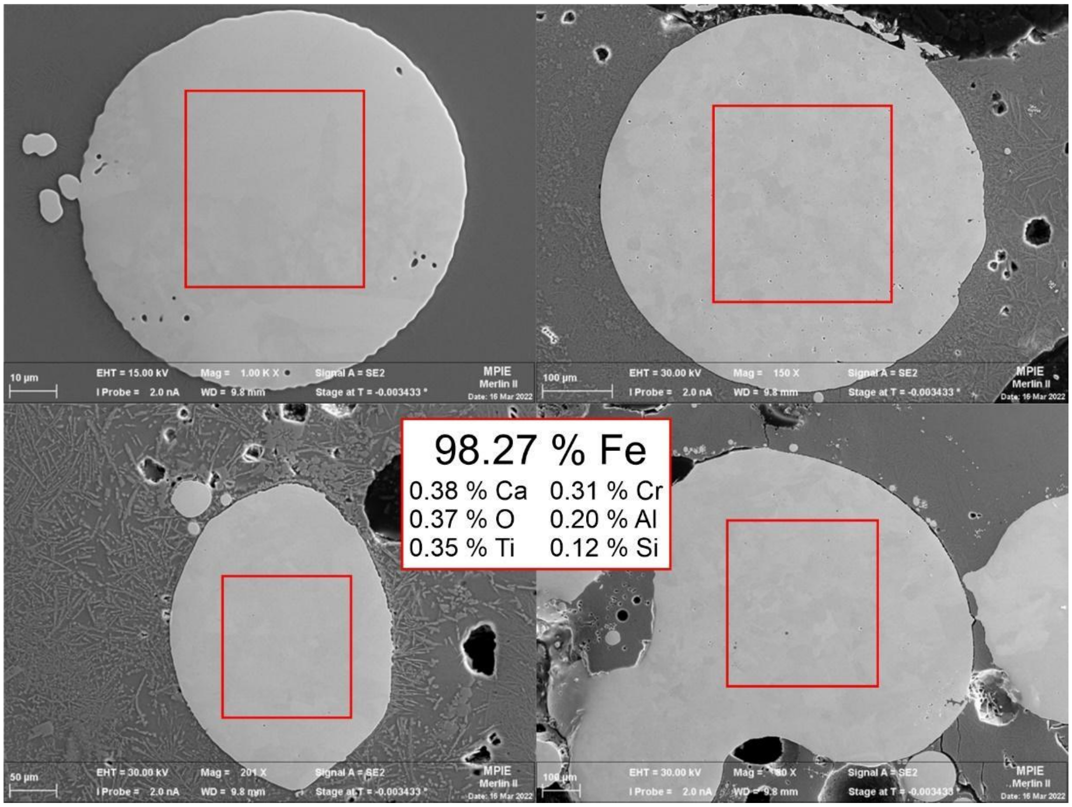

Fig. S2: **Exemplar SEM micrographs of sub-mm-sized iron nodules and their average purity (in wt.%) determined with EDS**. The probed areas are marked with a red square for each nodule. The standard deviation (SD) for Fe is 0.55 wt.%. For Ca, O, Ti and Cr the SD is 0.15 wt.%, whereas for Al and Si the SD is 0.05 wt.%.

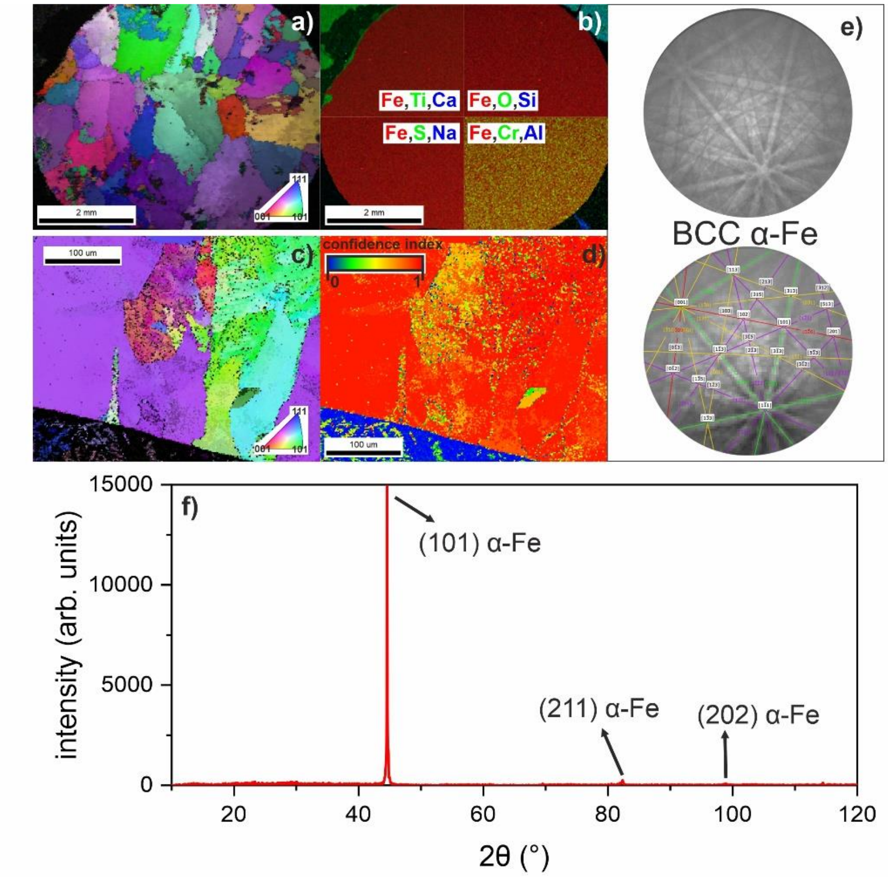

Fig. S3: **Microstructural and crystallographic aspects of extracted Fe nodules. (a)** Inverse pole figure (IPF) of an exemplary iron nodule extracted from the reduced samples. **(b)** Chemical maps of the iron nodule from (a) measured with energy-dispersive X-ray spectroscopy (EDX). The iron nodule is subdivided into 4 tricolour (red-green-blue) sections to present the chemical distribution of different elements (indicated in the middle part of the image for each section) within and around the iron nodule. **(c)** IPF of an enlarged region of the iron nodule with a part of the oxide in its surrounding (see lower left portion of image). **(d)** a colour-coded map of the confidence index ranged from 0 to 1 (blue to red) to indicate the matching of the indexing of the crystal structure with referenced BCC α-Fe crystal structure through Kikuchi pattern indexing. **(e)** Exemplar Kikuchi pattern obtained from the iron nodule showing a high matching with the BCC α-Fe, which is shown through the indexing of the same pattern below it. **(f)** X-ray diffraction (XRD) pattern of the iron nodule displaying single BCC α-Fe crystal structure.

Theoretical limits of Fe extraction

The theoretical (equilibrium) Fe yield over the course of hydrogen-based reduction was assessed with the aid of the ThermoCalc software. For this purpose, 15 g of molten red mud was deliberatively exposed to increasing amounts of a gas mixture of Ar-10%H_2. The system was kept at a constant temperature of 1850 ºC and at an absolute pressure of 1×10^5 Pa. Both equilibrium

and mass partitioning amongst the constituents were permitted, as described in the section "Thermodynamic calculations" in Methods. The obtained results are shown in Fig. S4. This figure reveals that the maximum amount of Fe that can be extracted under equilibrium is 2.67 g, as particularly evidenced by the corresponding inset (see "basicity = 0.43 (original red mud))" in Fig. S4a. Compared to the experimentally obtained value of 2.6 g Fe, we can infer that the hydrogen plasma reduction process used in this work proceeds at the thermodynamic limits, and the maximum amount of metallic Fe was indeed obtained for temperatures typically observed at plasma/melt reaction interfaces (see for example Ref.[44,45]).

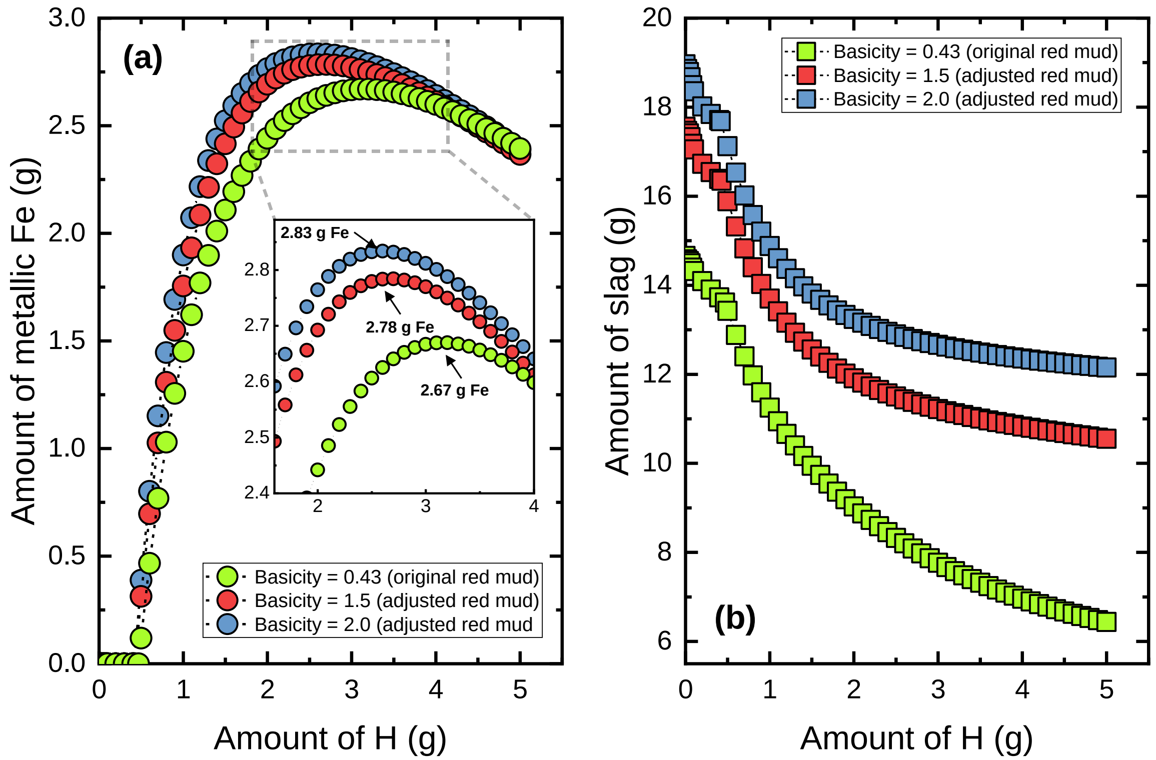

Fig. S4: Maximum amounts of metallic iron extracted from red mud in equilibrium conditions. (a) Equilibrium amounts of metallic Fe that can be extracted from the red mud investigated in this work (basicity = 0.43) in comparison with the corresponding Fe quantities for the red mud with adjusted basicity values of 1.5 and 2.0 (through the addition of CaO). (b) Equilibrium quantities of slag that is found in equilibrium with the Fe values documented in (a). Calculations conducted for a constant temperature of 1850°C at an absolute pressure of 1×10^5 Pa.

Based on this finding, we also evaluated the impact of the chemistry, namely, basicity of the red mud on the Fe yield through thermodynamic calculations. We considered as basicity the following ratio:

$$\text{Basicity} = \frac{(\text{wt.}\%\ \text{MgO} + \text{wt.}\%\text{CaO})}{\text{wt.}\%\text{SiO}_2}$$

The red mud investigated in this work has MgO, CaO and SiO_2 contents of 0.19, 6.52 and 15.43%, respectively (see Fig. S1). Thus, resulting to a basicity of 0.43.

To adjust the basicity of the red mud – through the addition of lime (CaO), for example – to the conventionally accepted values of 1.5 and 2.0, the "adjusted" red mud should display final CaO mass percentages of 22.2 and 27.7 wt.%, respectively. This means that additional 2.8 g and 4.2 g of CaO should be added to the 15-g input red mud to be reduced. These numbers can be translated into an increase of the input material quantities by 19% and 28%.

Based on these considerations, thermodynamic calculations were also conducted for the red mud displaying adjusted basicity values, and the obtained results are also shown in Fig. S4. With the addition of CaO, the additional yield of Fe marginally increases by only 0.11 g and 0.16 g for basicity values of 1.5 and 2.0, respectively, as seen in Fig. S4a. Thus, a gain of only 4 and 6% can be achieved by correcting the basicity to 1.5 and 2.0, respectively.

At this point, we should consider that CaO is obtained through the decomposition of $CaCO_3$ ($CaCO_3 \rightarrow CaO + CO_2$). Therefore, to produce 2.84 g and 4.2 g of CaO to adjust the basicity of the red mud, the corresponding stoichiometric amounts of 2.35 g and 3.45 g of CO_2 will be emitted. These numbers can be translated into approximately 21 g of CO_2 per each gram of additional Fe to be extracted from red mud with basicity values corrected to 1.5 or 2.0.

Furthermore, the equilibrium amount of slags at the maximum Fe extraction will be 7.57 g, 11.38 g and 12.84 g for the original red mud, and for the variants with basicity of 1.5 and 2.0, respectively. This means that 58% and 70% more slag must be handled at the end of the process. The composition of the slags at the maximum Fe extraction is shown in Table S2.

Table S2. Composition of the slag portions at the maximum Fe extraction, as obtained via thermodynamic calculations.

Composition of slag at maximum Fe extraction (wt.%)	Al	Ca	Fe	O	Si	Ti
Basicity = 0.43	13.46	10.56	10.41	39.94	10.22	14.90
Basicity = 1.5	8.96	24.87	7.54	38.23	10.26	9.93
Basicity = 2.0	7.94	29.59	6.58	37.43	9.48	8.80

The chemical composition of these slags evidences that the addition of CaO dilutes the important element Ti and others such as Sc, Y, etc. (the latter were not included in the thermodynamic calculations for the sake of simplicity), a fact that can potentially make their post-processing recovery more difficult. Therefore, the adjustment of the basicity of red mud prior to hydrogen plasma reduction might not be an attractive strategy in this work, because the hydrogen-based reduction of the original red mud proceeds near the thermodynamic equilibrium for temperatures typically found at reaction interfaces, and the addition of lime does not substantially enhance the Fe yield. On the contrary, it might 1) turn our process economically inviable due to the very marginal additional amounts of Fe that can be additionally extracted when compared with the ones obtained from the original red mud; 2) turn the process polluting via the indirect emissions of CO_2 with a high carbon footprint of 21 g CO_2 / g of additional Fe; (3) increase the amount of material to be processed before and after the process, demanding more energy; and (4) dilute important secondary elements in the slag, making their later recovery more difficult.

Fluid dynamics and implications on upscaling

In contrast to the hydrogen plasma reduction of pure hematite, where the produced liquid iron tends to sink to the bottom of the processed volume without any regular shape[15], iron produced from the red mud using the same process assumes a globular form. This fact indicates a strong influence of the oxide species originally contained in the red mud to form melts with different viscosities[55]. However, we found that, despite such complexity, the reduction efficiency seems not to be affected. On the contrary, the direct contact of the arc with the liquid induces a strong rotational stirring effect that creates a torus-like flow pattern which supports continuous mass transport of lightweight unreduced material to the reaction front between the reducing plasma and the liquid, where the reduction occurs. In combination with the hydrodynamic aspects of the system, the immiscibility between the reduced liquid and the remaining oxidic portions allows for their complete mass separation, as evidenced by the spherical metallic Fe nodules at the bottom of the samples.

This phenomenon can also occur within an up-scaled system, such as in an EAF, as the mass separation through the immiscibility of oxide and metallic liquids is mainly driven by their physical and chemical properties (viscosity and mass density), allowing for tapping liquid Fe from the bottom of the reactor after the arc is switched off. However, to sustain the high conversion

degrees over the process, sufficient hydrodynamic conditions are necessary to promote circulation of mass inside an industrial EAF. This is of particular importance to induce mass transport of the unreduced oxidic liquid towards the reaction interface between the hydrogen plasma arc and the melt (i.e., the region where the redox chemical reactions preferably take place). In general, a single electrode DC EAF yields efficient mixing due to the strong stirring induced by the electromagnetic forces from the current passing through the melt as well as immense thermal gradients, a condition similar to that in our experiments, thus maintaining the transport of mass towards the reaction interface[44]. The stirring effects in such furnaces can be further optimized with short arcs ignited at high currents[45]. However, when considering the processing of red mud in three-electrode AC EAFs, the electromagnetic forces in the melt that create mass circulation can be smaller because the alternating electric current does not travel through the melt, but flows from one electrode through the charged material to the neighbour electrode[44,56].

The short interaction between electric arcs and melts in AC EAF permits the creation of convection lines in the liquid which are mainly driven by gradients of temperature – i.e., the temperatures of the melt immediately underneath the arc are higher (e.g., 2100°C) than those at portions of the liquid closer to the reactor's wall (e.g., 1560°C)[45]. However, convection induced by thermal gradients in AC EAF might not be sufficient to maintain the transport of molten red mud to the reaction interface due to the strong friction between slag and metallic liquid and the high viscosity of the oxidic melts. Under such conditions, one could consider additional strategies to enhance mass circulation[44] such as (1) adequate arc length. Long arcs are suggested to increase the velocity of the melt flow[45]. However, the optimisation of arcs in three-electrodes AC EAF must consider the Lorentz forces that deflect them apart, thus directly impacting on their ideal length[57]; (2) Another option is gas injection at the bottom of the furnaces to create localized vortices that are capable of increasing the turbulence of the melt. In this sense, one might also consider the injection of gas mixtures containing a fraction of reducing hydrogen to promote further chemical reactions within the melt[58]. (3) Also, magnetic stirring at the bottom of the EAF is an option which can enhance the melt flow velocities by a factor of 10, also creating turbulences throughout the volume[59]. At this point, it is important to recall that most of these observations come from modelling works for hydrodynamics aspects in AC EAF conventionally used to melt steel scrap and direct reduced iron (DRI)[44]. It is important that further modelling includes instead the red

mud's physical properties and the coexistence of substantial quantities of two liquids (slag and iron) for a better quantitative and qualitative descriptions of the material dynamics.

Two further aspects become important when considering the scalability of red mud processing in EAF, viz. the concentration of hydrogen in the input gas and the electrode material. It is likely that hydrogen will be introduced into an industrial EAF under a constant gas flow. In this sense, not only an appropriate balance between the partial pressure of hydrogen in the input gas and the feeding flow is important for appropriate reduction kinetics, but also the mechanism for gas insertion into the furnace. It might be that gas mixtures containing hydrogen partial pressures above 10% will be preferred, e.g., 60%Ar-40%H_2[59] due to the large volumes of red mud to be processed. Also, introducing hydrogen through hollow electrodes (probably made of graphite[60]) would force a downward flow of gas together with the electric arc, thus enhancing H_2 dissociation into H plasma species (via the collision of H_2 with electrons).

In our work, the sample processed for 15 min with considerably higher input energy (800 A) resulted in a lower yield of the metallic Fe (approximately 0.5 g less than that for the 10 min case). For this, the hypothesis is that the higher input power results in much more intense and turbulent flow patterns as well as the destabilisation of the self-formed slag layers that cannot act as a protection to the underlying material containing the Fe. A similar process occurs in EAF for steel production, when the slag is destabilized through excessive gas blowing and/or overheating that causes increased iron losses through additional Fe enrichment of the slag and direct metal evaporation[61,62]. Such increased losses at higher power indicate that the process has to be optimized from the perspective of input power in order to minimize the losses of converted material, whilst sustaining high conversion rates and effectiveness through moderate turbulent flow.

Technoeconomic assessment

The proposed processing of red mud with hydrogen plasma is from a technoeconomic point of view a relatively straightforward process, since for the reduction, melting and finalisation of the final red mud products, a single EAF is required. To assess the economic viability, a model for a typical 100-ton EAF with its inputs and outputs is taken as a basis for the analysis, Fig. S5a. The main assumptions and parameters for the EAF operation were extracted from Ref.[32]. The ensemble of the individual parameters as well as the general calculation of individual inputs and outputs are described in the following supplementary section "Technoeconomic assessment calculations". To

assess the total cost of red mud processing per ton of input red mud, the operational expenditures (OPEX) and capital expenditures (COPEX) of the whole process were ranged to a single 100-ton processing of red mud, which was assessed based on the yearly production capabilities and operational costs of a standard EAF. For the COPEX, a 20-year lifetime of the EAF is assumed. The capital outcome is further evaluated in relation to the fraction of hematite originally contained in the input red mud, as this directly impacts the quantities of pure iron to be tapped from EAF. To present the scales of the individual inputs and outputs of the process, exemplar values for the case of red mud with 30 wt.% hematite are given in Fig. S5a. For the evaluation of the costs of the main consumables, hydrogen and electricity, a general mean price is assumed that does not only include sustainable production sources but less costly blue and grey hydrogen are considered as well, priced 3.3 EUR/kg and 50 EUR/MWh, respectively. Additionally, the red mud that was used in this work came from dried feedstock, i.e., the red mud was already pre-dried. However, it must be considered that the red mud can initially have on average about 30 wt.% water content, when stored in dry disposal ponds, depending on its location and processing history[47]. Thus, the additional median power consumption of 302 kWh needs to be considered per ton of dry red mud, translating to an additional average cost of 15.1 EUR per ton of dried red mud to be processed via hydrogen plasma reduction (for more details see supplementary section "Technoeconomic assessment calculations").

As can be seen in Fig. S5b, the final costs of red mud processing can have a positive capital turnaround when the red mud contains at least 50 wt.% of hematite (see green coloured region in Fig. S5b). For the first analysis, the costs associated with the landfill disposal of red mud were not considered. The result reveals that this turnaround is already possible by the hydrogen plasma process itself without considering the capital currently expended to dispose red mud. When also adding the costs for red mud disposal, the compensated capital yield is positive already at hematite mass fractions of at least 30 wt.% (see blue coloured region in Fig. S5b), meaning that the process can be financially viable also for red muds with lower hematite contents. However, below 30 wt.% hematite, the red mud processing is not financially viable, which goes in hand with the lower iron yield originating from the smaller iron quantities available in the initial red mud.

To put the new process also in an economical perspective relative to the processing of commercially available hematite ores, calculations with high-grade (62 wt.% Fe) and low-grade (45 wt.% Fe) hematite ores were performed, as documented in the supplementary section

"Hematite ore calculations". The calculations project that the process with hematite ores is financially more viable, reaching positive financial outcome of around 70 EUR/t and 132 EUR/t for the low-grade and high-grade hematite, respectively. When the costs for the ores are included (extraction, beneficiation, pelletizing, etc.), the financial viability of the process remains positive at 20 EUR/t and 22 EUR/t for the low-grade and high-grade hematite ores, respectively. These rather comparable numbers indicate that the processing of the red mud material with higher hematite contents provide a similar financial outcome as the processing of hematite ores, providing further support to the economic and environmental positive outcome of red mud processing with hydrogen plasma. An additional effect that has to be taken into consideration however are the rapidly rising costs for red mud deposition, an effect that could render the processing of red mud even altogether commercially more attractive than hematite processing.

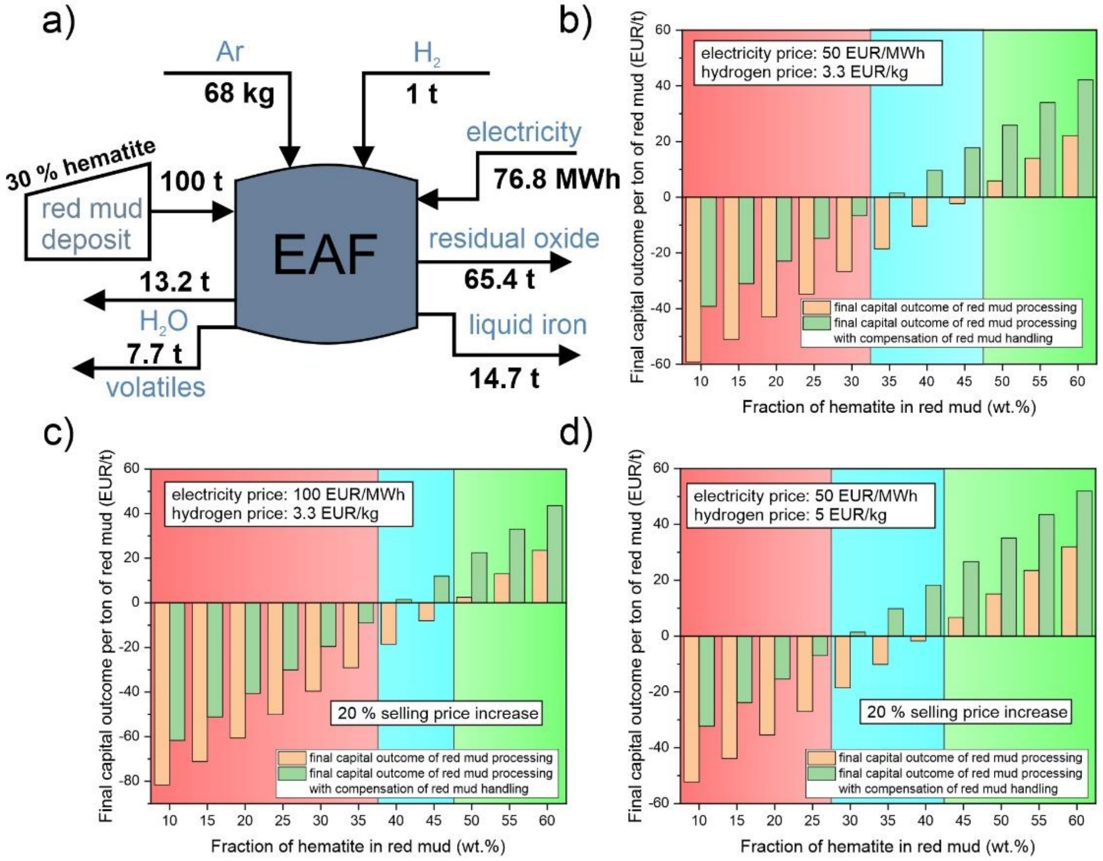

Fig. S5: (a) Model of the electric arc furnace (EAF) with individual inputs and outputs for the processing of red mud with a 10% hydrogen plasma. The indicated values for individual inputs and outputs provide an example for red mud with 30 wt.% hematite. **(b)-(d)** Calculated final processing costs/revenue with plasma processing of red mud material with different fraction of

hematite based on **(b)** nominal energy and hydrogen costs **(c)** increased electricity costs and **(d)** pure green hydrogen. For **(c)** and **(d)** the calculations include an increased selling price of products by 20%. The different coloured regions in **(b)-(d)** correspond to the different capital yield of the red mud processing (green: positive yield; blue: negative, but positive through compensation of red mud handling costs; red: negative yield, even with compensation of red mud handling costs).

The financial viability of this process (hydrogen plasma reduction) is strongly dependent on the cost of operation, namely the electricity and hydrogen prices as well as the market price of the final products, i.e. raw iron and residual oxide. To display the impact of utilising green hydrogen (cost of 5 EUR/kg) and higher electric energy costs (100 EUR/MWh), the final costs for extracting Fe from red mud via hydrogen plasma are recalculated as presented in Fig. S6 for each case separately. The trends indicate that the increased electricity price has a stronger negative impact on the final financial outcome of the process with increasing hematite fraction compared to the impact of the hydrogen price. However, with compensation of the red mud handling costs, the process is financially viable with hematite fractions of at least 55 wt.% and 60 wt.% for the higher hydrogen and electricity costs, respectively.

The apparent negative trend of the financial outcome indicates a discrepancy originating from the fixed price of the raw Fe and the remaining oxides that can be used in the cement industry. It should be clear that with increasing energy prices, the comparative prices of raw iron and cement oxides from other conventional processes would also increase, meaning that the process presented here would result in a comparatively positive financial outcome and positive trend with increasing hematite fraction. To present the strong influence of price correction on the financial outcome, additional data with 20% selling price increase (reasonably assumed based on price fluctuations[48]) is provided in Fig. S5 for the cases of higher electricity costs (Fig. S5c) and higher hydrogen costs (Fig. S5d). The new data presents a much stronger financial viability of the process that scales positively with hematite fraction that follows closely the trend from the initial calculation presented in Fig. S5b. As can be seen, the red mud processing is financially viable on its own from 45 to 50 wt.% hematite (see green region in Fig. S5c-d), whereas the viability with the addition of the compensation of the red mud waste management improves viability down to cases with hematite fractions of only 25 to 30 wt.% (see blue region in Fig. S5c-d and note the differences between the two cases of higher electricity cost and higher hydrogen cost).

The process indicates a positive financial outcome for most commercially available red mud wastes (average hematite fraction of around 30-45 wt.%). It should be noted that currently the

processing of iron ores for steel production does not use 100 % green hydrogen nor use electricity with high costs of 100 EUR/MWh[49,50]. These two conditions were only considered here to set the very extreme cases for the calculations. Furthermore, with hydrogen plasma, the CO_2 emission associated with the liquid iron produced is much lower compared to the conventionally utilized pathways of crude iron production (blast furnace and basic oxygen converter integrated route), because in our process H_2O is the redox product and not CO_2. This also contributes to an improved positive yield of the process especially in view of the rising taxation of CO_2 emissions[50].

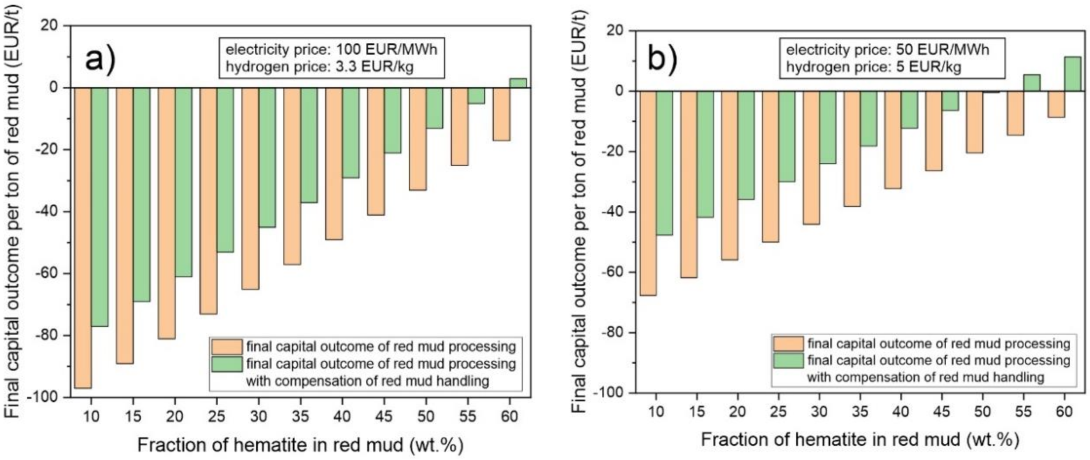

Fig. S6: **Calculated final processing costs/revenue with processing of red mud material with different fraction of hematite.** Calculations based on **(a)** increased electricity costs and nominal hydrogen costs **(b)** nominal electricity costs and increase hydrogen costs associated to utilization of green hydrogen.

A corresponding life cycle analysis (LCA) of the process can be performed using the reference calculations and considerations for red mud waste management provided by Joyce and Björklund[31]. With the values related to climate change due to CO_2 emissions, we can conclude that the hydrogen plasma processing of red mud has a positive net environmental balance with -601 kg CO_2 per 1 ton of red mud. This is possible due to the reduced CO_2 emission from the avoidance of carbon-based smelting that is assumed in the calculations of Joyce and Björklund[51]. When translating the metallisation of the process to the current market scenario, our reduction method could thus transform the existing 4 billion tons of red mud into 748 to 942 million tons of green steel (depending on the hematite content within the red mud, which normally ranges from 30 to 60 wt.%), thereby providing opportunities to cut 1.50 to 1.88 billion tons of CO_2 emissions associated with the currently used primary synthesis of iron. When also considering the contribution from the

red mud in the LCA, the environmental net outcome could be reduced by as much as 2.4 billion tons of CO_2 emission, when the current global red mud is utilized in full.

As a final remark, we can stress that the advantage of hydrogen plasma-based reduction process is by itself nearly free of direct CO_2 emissions and can be powered through renewable electric energy, making it an ideal process from the standpoint of sustainability. Submitting red mud to hydrogen plasma-based reduction to obtain Fe might not entirely solve all problems of CO_2 emission from the steel industry (because indeed the total Fe production is much larger), but it allows for tackling in the first place the unresolved problem of red mud accumulation, replacing the current harmful deposition method by its transformation into valuable feedstock that can be exploited. This can thus help to avoid its ever-growing dumping into the environment, an approach which is both essential from a sustainability perspective but potentially also attractive from an economical point of view, in view of the ever-increasing deposition costs. However, we should also consider that 35 million tons of iron produced via the conventional primary synthesis (i.e., through the blast furnace and oxygen converter route) would be accompanied by approximately 70 million tons CO_2 emissions per year. In the current scenario of global warming and its grim consequences for the planet, any possible avenue towards large-scale cutting of CO_2 emissions is essential.

Technoeconomic assessment calculations

Table S3: List of parameters for technoeconomic assessment of red mud processing

Parameter Acronym	Description	Value [units]
$\mathbf{mm_{Fe}}$	Molecular mass of iron	0.056 [kg/mol]
$\mathbf{mm_{O}}$	Molecular mass of oxygen	0.016 [kg/mol]
$\mathbf{mm_{H}}$	Molecular mass of hydrogen	0.001 [kg/mol]
$\mathbf{mm_{Fe2O3}}$	Molecular mass of hematite	0.16 [kg/mol]
$\mathbf{H_{Fe2O3}}$	Reaction enthalpy of hematite reaction with hydrogen gas to form iron and water	99500 [J/mol]
$\mathbf{WF_{evaporation}}$	Total weight fraction of evaporation	18 [wt.%]
**ME**	Metallization efficiency	70 [%]
$\boldsymbol{\rho_{Ar}}$	Gas density of argon gas in ambient environment	1.69 [kg/m^3]

$\mathbf{V_{EAF}}$	Volume of gas part of a standard 100 t furnace (assumed dimensions of 6 m diameter and 1.5 m height of the gas compartment)	42.41 [m^3]
$\mathbf{PR_{Ar}}$	Price of argon gas	0.931 [EUR/kg]
$\mathbf{PR_{H}}$	Price of hydrogen gas	3.30 [EUR/kg]
$\mathbf{PR_{EL}}$	Price of electricity	50 [EUR/MWh]
$\mathbf{PR_{L}}$	Price of labor	53.2 [EUR/1.3 trm^+]
$\mathbf{CAPEX_{EAF}}$	Capital expenses of a standard EAF with a service time of 20 years	184 [EUR/t]
$\mathbf{PR_{alloy}}$	Price of alloys for EAF	1777 [EUR/t]
$\mathbf{C_{alloy}}$	Consumption of alloys for EAF	7.77 [kg/trm^+]
$\mathbf{PR_{electrodes}}$	Price of electrodes	4000 [EUR/t]
$\mathbf{C_{electrodes}}$	Consumption of electrodes	1.415 [kg/trm^+]
$\mathbf{PR_{maintenance}}$	Cost of maintenance	3 % of CAPEX
$\mathbf{SP_{iron}}$	Selling price of raw iron	600 [EUR/t]
$\mathbf{SP_{oxides}}$	Selling price of residual oxides	60 [EUR/t]

*tli=ton of liquid iron; $^+$trm=ton of red mud

The values in Table S3 were extracted based on values provided by Vogl et al.[32] The values ranged based on final produced iron weight were recalculated based on equivalent oxide (hematite mass) used in regular EAF production of iron and referenced to the weight of processed red mud. This way the translation to the initial red mud weight was established. Furthermore, the production variables defined per year were recalculated based on the initial weight of oxides and ranged on the common yearly production yield of a typical 100 t EAF. All the required data was provided also in the work of Vogl et al. [32] The market price of raw iron and residual oxides was extracted from the reference of Balemenous et al.[33], which discusses the possible utilization and pricing of red mud products.

Calculation of individual parameters:

Produced amount of liquid iron M_{li} per 100 t per weight fraction of hematite in red mud WF_{Fe2O3}:

$$M_{li} = \frac{mm_{Fe}}{mm_{Fe2O3}} \cdot 2 \cdot WF_{Fe2O3} \cdot ME \cdot 100000$$

Produced amount of residual oxides M_{ro} per 100 t per weight fraction of hematite in red mud WF_{Fe2O3}:

$$M_{ro} = \left(100 - WF_{evaporation}\right) \cdot 100000$$

Energy consumption for reduction:

$$E_{reduction} = \frac{mm_{Fe2O3}}{2} \cdot H_{Fe2O3}$$

Energy consumption for heating of red mud is calculated to the temperature of 1620 °C, which is considered as the melting temperature of red mud[34,52] based on:

$$E_{heat} = Cp_{red\ mud} \cdot 100\ t \cdot 1600\ K$$

The specific heat capacity of the red mud was assumed to be 1.31 J/g K, as given by Wu et al.[35]

Energy consumed for the melting of the red mud at the melting temperature is calculated from the melting energies of the individual major oxide phases, namely Fe_2O_3, TiO_2, SiO_2, Al_2O_3, CaO and Na_2O. Since the non-hematite oxides have large melting energies, the highest energy consumption with 10 % hematite is applied for all cases. For hematite, the form magnetite (Fe_3O_4) was used for calculating them melting temperature due to the thermal decomposition of hematite to magnetite at temperatures slightly below the melting temperature.

The list of individual melting energies is provided in Table S4:

Table S4: List of melting energies *q* for individual major oxides of red mud. Values were obtained from Ref.[36]

Oxide	Melting energy [J/g]
**Fe_2O_3/Fe_3O_4**	596
**TiO_2**	944
**SiO_2**	159.8
**Al_2O_3**	1093
**Na_2O**	774.5
**CaO**	1427

$$E_{melt} = \sum q_{oxide} \cdot W_{oxide}$$

At which, the q_{oxide} is the melting energy of an individual oxide and the W_{oxide} is the mass fraction of the oxide in the red mud. The sum of both heating and melting energy of the red mud gives us the total energy consumed EAF through thermal processing of the red mud (E_{h+m}):

$$E_{h+m} = E_{heat} + E_{melt}$$

Total power consumption P_{total} (per 100 t of red mud) dependent on WF_{Fe2O3}:

$$P_{total}[kWh] = \frac{E_{h+m}}{3600} + \frac{WF_{Fe2O3} \cdot E_{reduction} \cdot ME}{3600}$$

For above equation the operation time of the whole process is assumed to be 1 h.

The argon consumption is calculated based on the whole volume of the gas compartment of a standard 100 t EAF with 6 m diameter. It is assumed that most of the Ar gas remains within the EAF throughout the red mud processing. Since a 10 % H_2 gas was used in our experiments, the same ratio is used for the calculations, meaning 90 % of the EAF volume is filled with Ar gas. A 5 % loss of the gas is additionally calculated. The total argon amount M_{Ar} needed for the process is:

$$M_{Ar} = V_{EAF} \cdot 0.9 \cdot \rho_{Ar} \cdot 1.05$$

Total hydrogen consumption:

The total hydrogen gas consumption is the sum of the consumed hydrogen gas from the initial evaporation of the clay material and later by the actual reduction of the hematite with hydrogen (it is assumed that only hematite plays a crucial role in the reduction-based consumption of hydrogen). The calculation of these two contributions are as follows:

The hydrogen consumption from initial evaporation is extracted from the difference in the final weight between the sample treated with hydrogen for 1 min with the initial sample weight (13.248 g and 15 g, respectively) ΔM, Fig. 5(b) of the manuscript. Thus, the weight difference between the samples can be correlated to the reduced weight of the material with hydrogen. Since the material consists of approximately 35 wt.% O (Fig. 1d), it is assumed that the evaporated material weight is also only reacting in terms of formation of water from oxygen and hydrogen. Additionally, the red mud samples hold approx. 4.2 wt.% of water and 2.2 % clay material volatiles that are additionally disregarded in the calculation, as these are assumed to not consume hydrogen. As a result, the consumption of hydrogen after evaporation $M_{H,evap}$ per 100 t is:

$$M_{H,evap} = \frac{\Delta M - 0.064 * 15}{15} \cdot 0.35 \cdot \frac{mm_H}{mm_O} \cdot 2 \cdot 100000$$

Whereas the mass of hydrogen with reduction of hematite $M_{H,reduct}$ per 100 t is:

$$M_{H,reduct} = \frac{mm_H}{mm_{Fe2O3}} \cdot 6 \cdot WF_{Fe2O3} \cdot ME \cdot 100000$$

Thus, the total hydrogen consumption M_H is:

$$M_H = M_{H,reduct} + M_{H,evap}$$

Cost calculations:

For the EAF, the capital expenses *CAPEX* are extracted from a conventional calculation for an EAF based on Vogl et al.[32] Thus, the CAPEX of the model 100 t EAF in our case is assumed to be:

$$CAPEX = CAPEX_{EAF} \cdot 100$$

The total operational expenses *OPEX* are separated into the processing costs $OPEX_{proc}$ that come from the actual gas and energy consumption during red mud processing and on the operational costs $OPEX_{oper}$ that include the labor, EAF alloy replacement and electrode consumption as well as maintenance costs. The $OPEX_{proc}$ is calculated as:

$$OPEX_{proc} = M_{Ar} \cdot PR_{Ar} + M_{H} \cdot PR_{H} + P_{total} \cdot PR_{EL}$$

Whereas the $OPEX_{oper}$ per 100 t is calculated as:

$$OPEX_{oper} = PR_{\mathrm{maintenance}} + PR_{L} \cdot 76.923 + PR_{alloy} \cdot C_{alloy} \cdot 10 + PR_{electrodes} \cdot C_{electrodes} \cdot 10$$

The total OPEX is thus:

$$OPEX = OPEX_{proc} + OPEX_{oper}$$

Selling value of final products:

The final selling values of the raw iron and residual oxides are designated based on the nominal values of the current iron prices (about 600 EUR/t) and prices of oxides for utilization in cement and construction industries. For the oxides it is assumed that they can be separated further into more valuable and less valuable oxides which yield on average value of about 60 EUR/t. The total selling value of the end products is thus:

$$SV = M_{li} \cdot SP_{iron} + M_{ro} \cdot SP_{oxides}$$

Assumption of disposal costs of red mud:

In literature, the safe disposal costs of red mud PR_{RM} is evaluated at about 10 EUR/ton, which reaches about 2 % of alumina market price[5]. However, such cost estimate has been delivered more than 15 years ago and cannot be considered a real indicator of today's costs related to red mud disposal. Especially, such an estimate might be outdated due to the increased environmental costs and taxes of solid waste management that are continuously rising with time to accommodate

the improved and stricter protection of the environment. Although exact numbers on increased costs and taxes for red mud management could not be obtained, a comparative note can be assumed based on standard solid waste landfill taxes. The example for Industrial Landfill Levy in Victorian municipality in Australia[52] clearly presents that since 2005 the taxes on solid waste management have increased nearly 6-fold meaning that the costs of proper waste management have grown considerably within the last 15 years. In this case we can assume that the costs of proper disposal PR_{RM} could be as high as 20 EUR/ton, if we assume that the processing cost mainly originate in adaptation of current dam systems, increased transportation and management costs as well as inflation over a period of 15 years.

Financial outcome *FO* of red mud processing with hydrogen plasma based EAF reduction:

The final financial outcome of the red mud processing per ton of red mud is the sum of all expenses and selling inflow of the process:

$$FO = (SV - OPEX - CAPEX)/100$$

The financial outcome of the process compensated by the price of red mud handling for disposal FO_{comp} is calculated as:

$$FO_{comp} = FO + PR_{RM}$$

Calculation of energy required for red mud drying

The red mud that is commonly disposed as dry cake has on average a 30 wt.% water content, which needs to be removed down to 4.2 wt. % before utilization in the reduction process as in these specific experiments. To commensurately achieve the final dry red mud feedstock of 1 ton, the calculations are assumed with drying of 1.35 tons of wet red mud. Thus 350 kg of water is considered as mass of water needed to be evaporated from the initial wet stockpiled red mud. The calculation of the power for the drying of the red mud is assessed based on reference values of the required energy to dry 1 ton of water from a sludge using conventional industrial scale sludge dryers that can range from 682 to 1045 kWh per ton of evaporated water[37]. As such, the consumed power for the pre-drying of red mud is from 238.7 kWh to 365.8 kWh, depending on the selected

technique and apparatus[37]. With the considered energy price of 50 EUR/MWh, the price of drying would range from 11.9 to 18.3 EUR per ton of dried red mud. For the purpose of integrating a single value for the drying contribution to the final cost of red mud processing, the mean value of 15.1 EUR per ton of dried red mud is used in the calculations.

Hematite ore calculations:

For the purposes of comparing the costs of processing red mud with hematite ore, a commercial hematite ore with 62 wt.% of Fe is considered, which is valued at about 110 EUR/t[38]. Additionally, a low-grade iron ore with 45 wt.% of Fe[39], valued at 50 EUR/t is also considered in order to evaluate the financial outcome of an iron ore of similar Fe content as that of some red muds. For the calculation of the metallization efficiency for both low-grade and high-grade hematite ores, the extraction capability of 90 % is used to incorporate the high efficiency of the reduction process and iron losses due to evaporation as experimentally determined beforehand[53]. Since the additional oxides within hematite ores are mostly Si-based[54], the utilization of such residues is limited. For this reason, the residual oxide residue is valued at only 10 EUR/t to envelope the limited applicability of such oxide residues[33].

40. Qaidi, S. M. A. et al. Sustainable utilization of red mud waste (bauxite residue) and slag for the production of geopolymer composites: A review. Case Studies in Construction Materials **16**, e00994 (2022).

41. Rao Borra, C. et al. Smelting of Bauxite Residue (Red Mud) in View of Iron and Selective Rare Earths Recovery. Journal of Sustainable Metallurgy **2**, 28-37 (2016).

42. Sabat, K. C. & Murphy, A. B. Hydrogen Plasma Processing of Iron Ore. Metallurgical and Materials Transactions B: Process Metallurgy and Materials Processing Science **48**, 1561–1594 (2017).

43. Li, B., Sun, G., Li, S., Guo, H. & Guo, J. The Preparation of High-Purity Iron (99.987%) Employing a Process of Direct Reduction–Melting Separation–Slag Refining. Materials 2020, Vol. 13, Page 1839 **13**, 1839 (2020).

44. Odenthal, H. J. *et al.* Review on Modeling and Simulation of the Electric Arc Furnace (EAF). *Steel Res Int* **89**, 1700098 (2018).

45. Gonzalez, O. J. P., Ramírez-Argáez, M. A. & Conejo, A. N. Effect of Arc Length on Fluid Flow and Mixing Phenomena in AC Electric Arc Furnaces. *ISIJ International* **50**, 1–8 (2010).

46. Gudim, Y. A., Ovchinnikov, S. G. & Zinurov, I. Y. Metal losses during steelmaking in arc furnaces and methods for their decreasing. Russian Metallurgy (Metally) 2011 2011:6 **2011**, 495–498 (2011).

47. Patil, S. V. & Thorat, B. N. Mechanical dewatering of red mud. Sep Purif Technol **294**, 121157 (2022).

48. Pig Iron Price in Germany - 2023 - Charts and Tables - IndexBox. https://www.indexbox.io/search/pig-iron-price-germany/.

49. Wang, R. R., Zhao, Y. Q., Babich, A., Senk, D. & Fan, X. Y. Hydrogen direct reduction (H-DR) in steel industry—An overview of challenges and opportunities. Journal of Cleaner Production vol. 329 129797 Preprint at https://doi.org/10.1016/j.jclepro.2021.129797 (2021).

50. Comprehensive study: carbon taxes won't hamper the economy | Climate crisis | The Guardian. https://www.theguardian.com/environment/climate-consensus-97-per-cent/2018/jul/16/comprehensive-study-carbon-taxes-wont-hamper-the-economy.

51. Joyce, P. J. & Björklund, A. Using Life Cycle Thinking to Assess the Sustainability Benefits of Complex Valorization Pathways for Bauxite Residue. Journal of Sustainable Metallurgy **5**, 69–84 (2019).

52. Managing the Municipal and Industrial Landfill Levy | Victorian Auditor-General's Office. https://www.audit.vic.gov.au/report/managing-municipal-and-industrial-landfill-levy?section=.

53. Souza Filho, I. R., Ma, Y., Raabe, D. & Springer, H. Fundamentals of Green Steel Production: On the Role of Gas Pressure During Hydrogen Reduction of Iron Ores. JOM **75**, 2274–2286 (2023).

54. Dhawan, N., Manzoor, U. & Agrawal, S. Hydrogen reduction of low-grade banded iron ore. Miner Eng **187**, 107794 (2022).

55. Kaußen, F. & Friedrich, B. Reductive Smelting of Red Mud for Iron Recovery. Chemie Ingenieur Technik **87**, 1535–1542 (2015).

56. Cavaliere, P. Electric Arc Furnace: Most Efficient Technologies for Greenhouse Emissions Abatement. in Clean Ironmaking and Steelmaking Processes 303–375 (Springer, Cham, 2019). doi:10.1007/978-3-030-21209-4_6.

57. P. Frittella et al., 10th European Electric Steelmaking Conf., Graz, Austria 2012

58. Chen, S., Rong, Z., Li, J. & He, C. The Study of Three-Phase Numerical Simulation of Molten Bath in EAF Impinged by Supersonic Oxygen Jet. *Adv Mat Res* **479–481**, 1750–1756 (2012).

59. Arzpeyma, N., Widlund, O., Ersson, M. & Jönsson, P. Mathematical Modeling of Scrap Melting in an EAF Using Electromagnetic Stirring. *ISIJ International* **53**, 48–55 (2013).

60. Ernst, D., Zarl, M. A., Farkas, M. A. & Schenk, J. Effects of the Electrodes' Shape and Graphite Quality on the Arc Stability During Hydrogen Plasma Smelting Reduction of Iron Ores. *Steel Res Int* **94**, 2200818 (2023).

61. Gudim, Y. A., Ovchinnikov, S. G. & Zinurov, I. Y. Metal losses during steelmaking in arc furnaces and methods for their decreasing. Russian Metallurgy (Metally) **2011**, 495–498 (2011).

62. Serikov, V. A., Bikeev, R. A., Cherednichenko, M. v. & Cherednichenko, V. S. Metal loss and charge heating in the melt in an electric arc furnace. Russian Metallurgy (Metally) **2015**, 980–984 (2015).

63. Toxic Sludge in Hungary. https://earthobservatory.nasa.gov/images/46360/toxic-sludge-in-hungaryCopyright.

64. Mayes, W. M., Jarvis, A. P., Burke, I. T., Walton, M., Feigl, V., Klebercz, O., & Gruiz, K. Dispersal and Attenuation of Trace Contaminants Downstream of the Ajka Bauxite Residue (Red Mud) Depository Failure, Hungary. Environmental Science & Technology **45**, 12, 5147–5155 (2011).
